# Cytokinopathy with aberrant cytotoxic lymphocytes and pro-fibrotic myeloid response in SARS-CoV-2 mRNA vaccine-associated myocarditis

**DOI:** 10.1126/sciimmunol.adh3455

**Published:** 2023-05-05

**Authors:** Anis Barmada, Jon Klein, Anjali Ramaswamy, Nina N. Brodsky, Jillian R. Jaycox, Hassan Sheikha, Kate M. Jones, Victoria Habet, Melissa Campbell, Tomokazu S. Sumida, Amy Kontorovich, Dusan Bogunovic, Carlos R. Oliveira, Jeremy Steele, E. Kevin Hall, Mario Pena-Hernandez, Valter Monteiro, Carolina Lucas, Aaron M. Ring, Saad B. Omer, Akiko Iwasaki, Inci Yildirim, Carrie L. Lucas

**Affiliations:** 1 Department of Immunobiology, Yale University School of Medicine, New Haven, CT, USA; 2 Department of Pediatrics, Yale University School of Medicine, New Haven, CT, USA; 3 Department of Neurology, Yale University School of Medicine, New Haven, CT, USA; 4 The Zena and Michael A. Wiener Cardiovascular Institute; Mindich Child Health and Development Institute; Institute for Genomic Health, Icahn School of Medicine at Mount Sinai, New York, NY, USA; 5 Center for Inborn Errors of Immunity; Precision Immunology Institute; Mindich Child Health and Development Institute; Department of Pediatrics; Department of Microbiology, Icahn School of Medicine at Mount Sinai, New York, NY, USA; 6 Department of Medicine, Yale University School of Medicine, New Haven, CT, USA; 7 Epidemiology of Microbial Diseases, Yale School of Public Health, New Haven, CT, USA; 8 Yale Institute for Global Health, Yale University, New Haven, CT, USA; 9 Howard Hughes Medical Institute, Chevy Chase, MD, USA; 10 Yale Center for Infection and Immunity, Yale University, New Haven, CT, USA

## Abstract

Rare immune-mediated cardiac tissue inflammation can occur after vaccination, including after SARS-CoV-2 mRNA vaccines. However, the underlying immune cellular and molecular mechanisms driving this pathology remain poorly understood. Here, we investigated a cohort of patients who developed myocarditis and/or pericarditis with elevated troponin, B-type natriuretic peptide, and C-reactive protein levels as well as cardiac imaging abnormalities shortly after SARS-CoV-2 mRNA vaccination. Contrary to early hypotheses, patients did not demonstrate features of hypersensitivity myocarditis, nor did they have exaggerated SARS-CoV-2-specific or neutralizing antibody responses consistent with a hyperimmune humoral mechanism. We additionally found no evidence of cardiac-targeted autoantibodies. Instead, unbiased systematic immune serum profiling revealed elevations in circulating interleukins (IL-1β, IL-1RA, IL-15), chemokines (CCL4, CXCL1, CXCL10), and matrix metalloproteases (MMP1, MMP8, MMP9, TIMP1). Subsequent deep immune profiling using single-cell RNA and repertoire sequencing of peripheral blood mononuclear cells during acute disease revealed expansion of activated CXCR3^+^ cytotoxic T cells and NK cells. Both lymphocyte populations phenotypically resembled cytokine-driven killer cells. Additionally, patients displayed signatures of inflammatory and profibrotic CCR2^+^CD163^+^ monocytes, coupled with elevated serum soluble CD163. These findings may be linked to the late gadolinium enhancement seen on cardiac MRI, which can persist for months after vaccination. Taken together, our results demonstrate upregulation in inflammatory cytokines and corresponding lymphocytes with tissue-damaging capabilities, suggesting a cytokine-dependent pathology, which may further be accompanied by macrophage-associated cardiac fibrosis. These findings likely rule out some previously proposed mechanisms of mRNA vaccine-associated myopericarditis and point to new ones with relevance to vaccine development and clinical care.

## ONE SENTENCE SUMMARY

Cytokine-driven killer lymphocytes and inflammatory monocytes characterize myopericarditis after SARS-CoV-2 mRNA vaccination.

## INTRODUCTION

Vaccination against severe acute respiratory syndrome coronavirus 2 (SARS-CoV-2) is one of the most effective public health interventions in combating the ongoing coronavirus disease 2019 (COVID-19) pandemic. The SARS-CoV-2 spike messenger ribonucleic acid (mRNA) vaccines have been found to be safe in international studies involving hundreds of thousands of individuals (1–4), although very rare cases of adverse events have been subsequently reported (5, 6). Notably, one such adverse event is inflammation of the heart—namely myocarditis, pericarditis, or a combination of the two (myopericarditis) (7–12). SARS-CoV-2 vaccine-associated myopericarditis has been reported to occur most frequently in adolescent and young adult males during the first week following the second dose of an mRNA vaccine (BNT162b2 or mRNA-1273) (2, 5, 13, 14), although it can also occur across demographic groups after a single or third (booster) dose of mRNA vaccination or following non-mRNA-based vaccines (5, 15–19). Various estimates of myopericarditis risk following vaccination have been reported (2, 7, 14, 17, 20–23), with most recent reports estimating an incidence of 0–35.9 and 0–10.9 cases per 100,000 for males and females, respectively, across age groups and mRNA vaccine cohorts (24). A study of vaccine-associated myopericarditis incidence from our own healthcare network (Yale New Haven Hospital) between January and May 2021 identified 8 cases from 24,673 individuals aged 16–25 (0.3%) given two doses of mRNA vaccine (25).

Given the difficulties associated with studying such rare cases, the etiology of vaccine-associated myopericarditis remains largely unknown. A mechanistic understanding of the underlying pathology and associated immune alterations, as well as potential long-term effects, is needed and will likely have broad significance with the rapidly expanding clinical applications of mRNA vaccines (26–28). Early hypotheses to explain mRNA vaccine-associated myopericarditis speculated that the SARS-CoV-2 spike protein, which was detected in the blood (29) and sparsely on cardiomyocytes (30) of patients in some studies, may induce cardiac-targeted autoantibodies through molecular mimicry, though this has so far not been supported by initial reports (31, 32). Alternative hypotheses suggested that hypersensitivity myocarditis may instead explain the pathology, primarily based on clinical presentation, rapid recovery, and marked increase in incidence following second doses of mRNA vaccines (11, 33). However, early case reports and cardiac biopsies from small numbers of patients were largely inconsistent with such pathology (9, 34–36). Biopsy reports showed an inflammatory infiltrate predominantly composed of macrophages and T lymphocytes, though scattered eosinophils, B cells, and plasma cells were additionally noted in some reports (37). Autoimmune myocarditis driven by T helper type 17 (Th17) responses is another possible mechanism (38, 39), though no evidence supporting such pathology was found in the limited number of patients profiled to date (32, 40). Other arguments proposed aberrant immune reactivity, both innate and adaptive, triggered by the mRNA and/or lipid nanoparticles (LNP) (13, 41, 42). Further, recent studies in two patients implicated a role for various inflammatory cytokines as well as NK and T lymphocytes (32, 40). Each of these proposed mechanisms can be influenced by age, sex, and genetic background, leading to increased incidence in certain subpopulations (13). To better understand the underlying pathology in SARS-CoV-2 LNP-mRNA vaccine-associated myopericarditis, we conducted a multimodal study of acute myocarditis and/or pericarditis patients using unbiased approaches that define the maladaptive immune signatures during disease.

## RESULTS

### Vaccine-associated myopericarditis patient cohort and clinical presentation

**Fig. 1:**
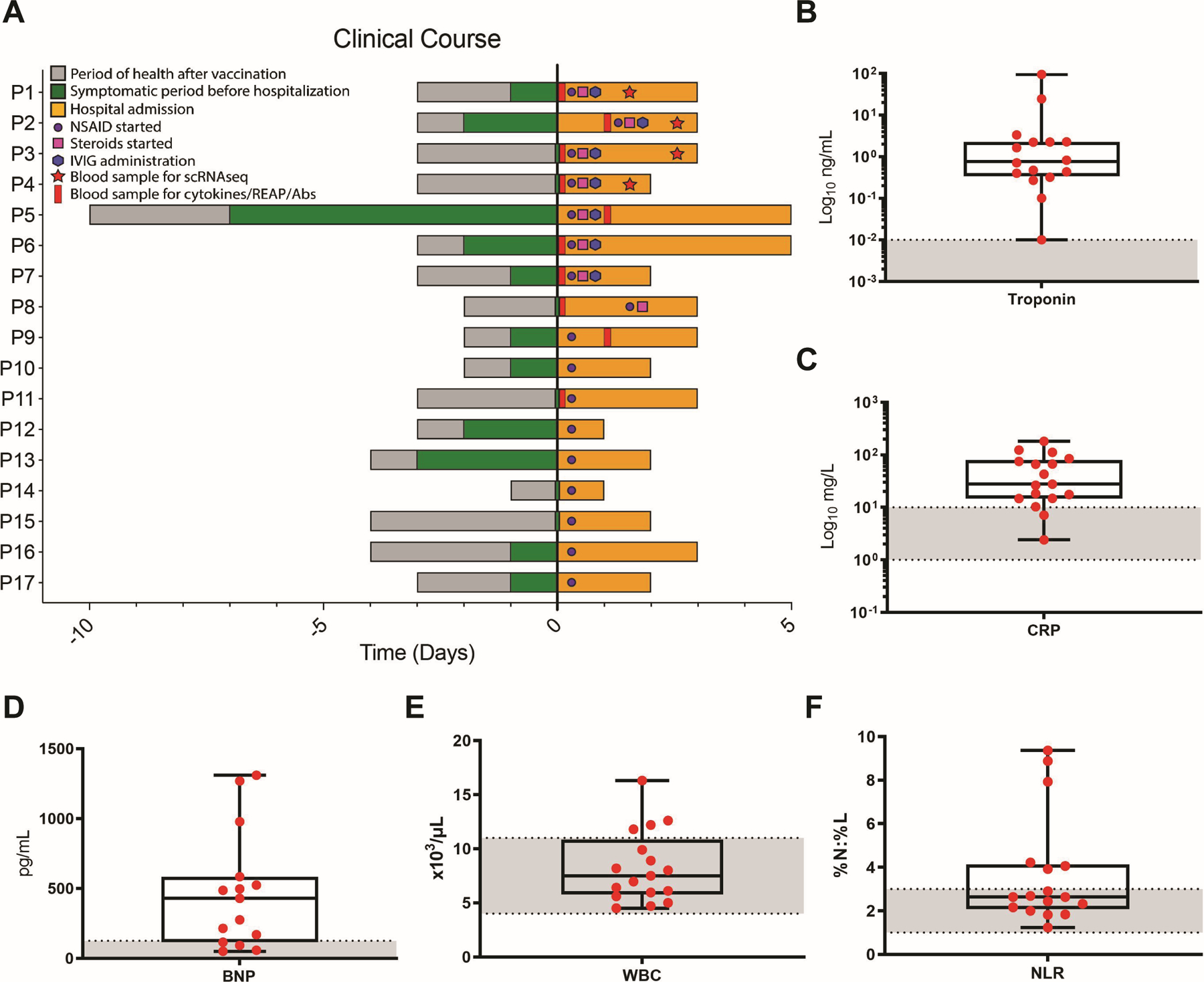
Clinical parameters of the SARS-CoV-2 vaccine-associated myopericarditis cohort. (**A**) Time course for patients showing the day of vaccine administration, symptom onset, treatment, and sample collection relative to hospital admission (Day 0). (**B** to **F**) Maximum values of selected blood markers in patients tested during hospital admission. Boxes depict the interquartile range (IQR), horizontal bars represent the median, whiskers extend to 1.5 × IQR, and red dots show the value of each patient. Dashed lines and gray area represent normal reference ranges as provided by the CLIA-certified Yale New Haven Hospital Department of Laboratory Medicine. Abbreviations: C-reactive protein (CRP), B-type natriuretic peptide (BNP), white blood cells (WBC), neutrophil-to-lymphocyte ratio (NLR).

Our clinical cohort consists of 23 vaccine-associated myocarditis and/or pericarditis patients. The cohort is predominately male (87%) with an average age of 16.9 ± 2.2 years (ranging from 13–21 years), in congruence with prior epidemiological reports (24). Patients had largely non-contributory past medical histories and were generally healthy before vaccination. Most patients had symptom onset one to four days after the second dose of the BNT162b2 mRNA vaccine (Fig. 1A, Table S1, and Table S2). Six patients either first experienced symptoms after a delay of > 7 days after vaccination (P18, P20, P22, and P23) or were incidentally positive for SARS-CoV-2 by polymerase chain reaction (PCR) testing upon hospital admission (P19 and P21) (Fig. S1A); these six patients were thus excluded from further analyses, though they potentially reflect the breadth of clinical presentations of vaccine-associated myopericarditis. Our remaining cohort of patients showed no evidence of recent prior SARS-CoV-2 infection, with antibodies to spike (S) protein but not to nucleocapsid (N) protein and negative nasopharyngeal swab RT-qPCR at hospital admission.

Symptom presentation was consistent with acute myocarditis and/or pericarditis, including chest pain, palpitations, fever, shortness of breath, headaches, myalgia, diaphoresis, fatigue, nausea/emesis, and/or congestion (Table S1). Laboratory testing in most patients during hospital admission demonstrated elevations in the maximum observed levels of troponin, C-reactive protein (CRP), and B-type natriuretic peptide (BNP) (Fig. 1B to D and Table S1), indicating acute systemic inflammation with direct myocardial injury. Some patients also had leukocytosis (Fig. 1E; for complete blood count differential, see Fig. S1C to K) and elevated neutrophil-to-lymphocyte ratio (NLR) (Fig. 1F), consistent with systemic inflammation. Notably, levels of eosinophils and basophils were within normal limits (Fig. S1F and G).

Electrocardiogram (ECG) performed at admission revealed abnormal findings ranging from nonspecific abnormalities to ST elevations and PR depressions consistent with pericarditis (Table S1). Echocardiogram findings demonstrated borderline low or abnormally reduced left ventricle ejection fraction (LVEF) in several patients, consistent with the diagnosis of myocarditis and/or pericarditis. Most patients further underwent cardiac magnetic resonance (CMR) imaging during acute hospitalization, revealing imaging abnormalities consistent with acute or subacute myocarditis and/or pericarditis (Table S1 and Fig. S1B).

Patients were treated with non-steroidal anti-inflammatory drugs (NSAIDs), and some also received steroids and intravenous immunoglobulin (IVIG) (Fig. 1A and Fig. S1A). Patients were discharged home with improving symptoms and clinical laboratory findings after one to six days. While patients showed rapid resolution of clinical symptoms with improved laboratory findings, most of them maintained some imaging abnormalities. These included late gadolinium enhancement (LGE) at longitudinal clinical follow-up at least two months after hospital discharge, as assessed by CMR (Table S1).

### Lack of evidence for potentially pathogenic antibodies in myopericarditis

**Fig. 2:**
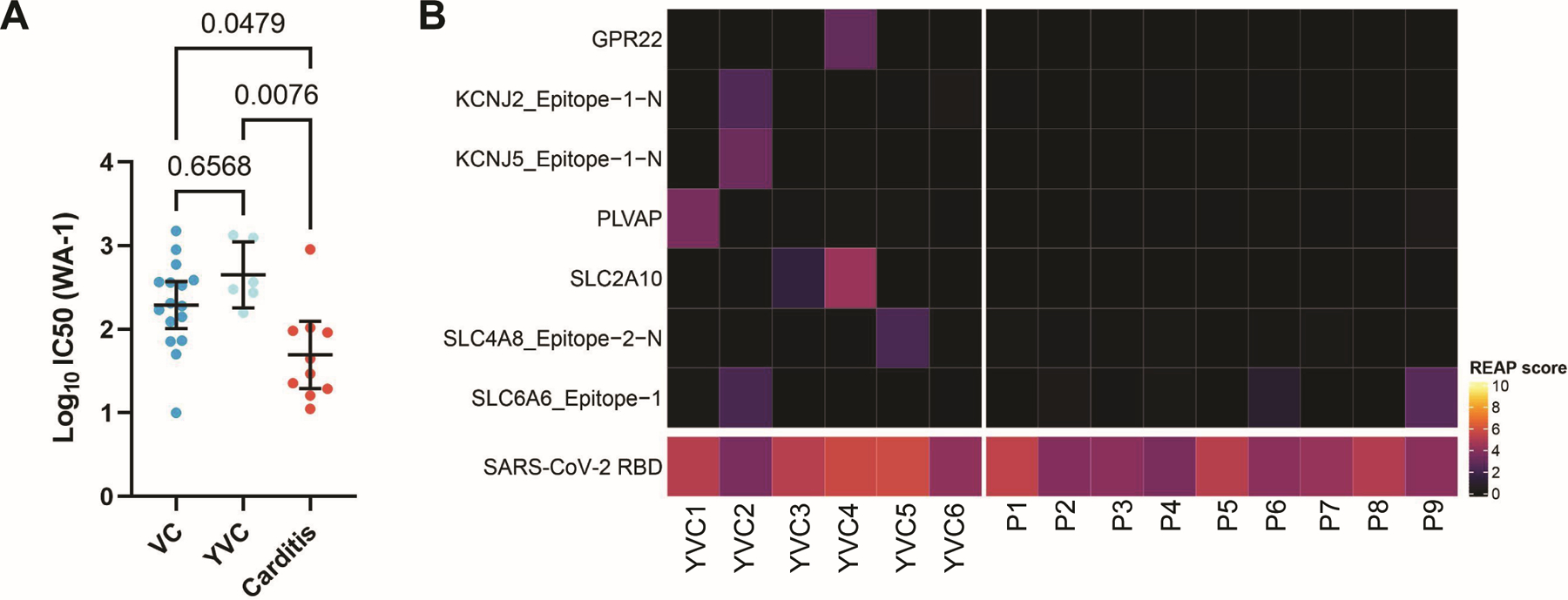
Myopericarditis patients mount humoral response to vaccination with absence of reactive autoantibodies. (**A**) IC_50_ estimates for SARS-CoV-2 neutralizing antibody titers against ancestral SARS-CoV-2 (WA-1) in patients (*n* = 10) compared to healthy vaccinated controls (VC, *n* = 16), in addition to those closer in age to patients where there is an increased risk for vaccine-associated myopericarditis (younger vaccinated controls (YVC), *n* = 6). Black bars denote group means and error bars represent 95% confidence intervals. Statistical significance was assessed using the Kruskal-Wallis test with Dunn’s correction for multiple comparisons. (**B**) Heat map of Rapid Extracellular Antigen Profiling (REAP) scores showing cardiac-related autoantibodies present in at least one donor (either patients or YVC), from a total of 526 antigens/epitopes defined by Gene Ontology (circulatory system process (GO:0003013) or heart contraction (GO:0060047)) or the Human Protein Atlas (heart tissue enriched/enhanced), across individual patients (*n* = 9) and YVC (*n* = 6). SARS-CoV-2 RBD antigen is used as a positive control in vaccinated individuals. Positive autoantibody reactivity is defined as REAP score ≥ 2, protein-wise z-score ≥ 1.96, and protein-wise mean < 0.5, with higher REAP scores correlating with higher antibody titer and/or abundance as validated previously (43, 44). Negative findings for all cardiac antigens/epitopes tested from the Human Protein Atlas (81 total) are further shown in Fig. S2D.

To first assess whether myopericarditis patients generate overexuberant humoral responses to vaccination, we performed enzyme-linked immunosorbent assay (ELISA) for SARS-CoV-2 specific S, S1 subunit, and receptor-binding domain (RBD) antibodies. Compared to healthy vaccinated controls (VC, *n* = 16), including those closer in age to patients where there is an increased risk for vaccine-associated myopericarditis (younger vaccinated controls (YVC), *n* = 6), myopericarditis patients (*n* = 9) had no evidence of enhanced anti-SARS-CoV-2 antibodies (Fig. S2A to C). Given the moderately reduced levels of SARS-CoV-2 specific antibodies observed in the patients, we next investigated whether the development of myopericarditis was associated with blunted generation of neutralizing antibody responses in these patients. We performed plaque reduction neutralization tests (PRNT_50_) and found a reduction in the estimated 50% serum inhibitory concentration (IC_50_) between patients and healthy vaccinated controls with no prior history of SARS-CoV-2 exposure (Fig. 2A), possibly owing to the differences in sample collection time relative to vaccination (3–11 days for patients vs. 7 days for controls). These results suggest that myopericarditis patients do not display enhanced SARS-CoV-2 specific and neutralizing antibody responses at the time of acute disease presentation, but rather show comparable or potentially blunted responses compared to healthy vaccinated controls.

To further interrogate whether myopericarditis patients generate self-targeted autoantibodies following vaccination, we employed Rapid Extracellular Antigen Profiling (REAP) to screen patients’ plasma for autoantibodies, as has been previously validated (43, 44). In total, autoantibodies recognizing 6,183 different human extracellular or secreted proteins/epitopes were assessed by REAP. Of these, we focused on 526 antigens/epitopes relevant to cardiac function, defined either as members of the Gene Ontology terms “circulatory system process” or “heart contraction,” or as “heart tissue enriched/enhanced” in the Human Protein Atlas. Our analysis showed no elevation in the frequency of targeting autoantibodies in myopericarditis patients compared to YVC (Fig. 2B and Fig. S2D). Of note, REAP applied to a separate control cohort of hospitalized COVID-19 patients (*n* = 60) showed positive signals for several of these cardiac-related autoantibodies, demonstrating the ability to detect such autoantibodies when present (Fig. S2E).

### Cytokinopathy revealed by unbiased analysis of serum proteins and immune cell subsets in myopericarditis

**Fig. 3:**
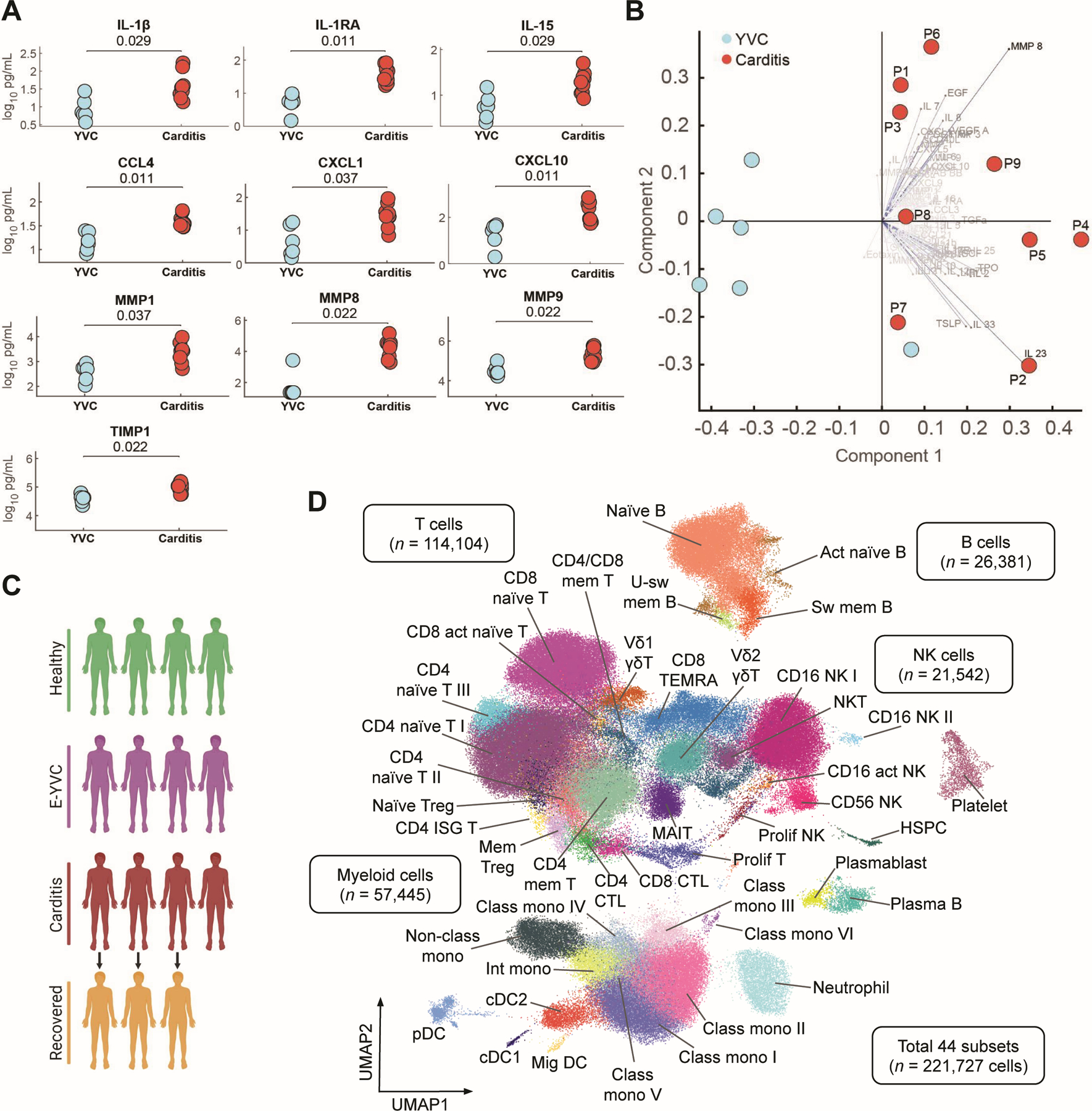
Elevation of immune cytokines in myopericarditis and identification of immune cell subsets. (**A**) Scatter plots of selected cytokines in patients (*n* = 9) and healthy younger vaccinated controls (YVC, *n* = 6). Statistical significance was determined using the unpaired two-sided Wilcoxon rank-sum test with Benjamini-Hochberg FDR correction for multiple comparisons. All additional serum proteins assayed are shown in Fig. S3 (**B**) Biplot of all serum proteins assayed (84 total) between patients and healthy YVC (see related principal component analysis (PCA) in Fig. S4, A and B). (**C**) Schematic diagram showing the cohort studied in subsequent scRNA-seq analyses, including pediatric male healthy donors (HD, *n* = 4), healthy early-young vaccinated controls (E-YVC, *n* = 4), acute myopericarditis patients (*n* = 4), and matched patients at follow-up/recovery (*n* = 3). (**D**) UMAP visualization of immune cell subsets identified from the cohort in (**C**), with additional four CITE-seq multisystem inflammatory syndrome in children (MIS-C) after SARS-CoV-2 samples included to refine cluster annotation using surface proteins. Cell subset abbreviations: activated (act), class-switched (sw), class-unswitched (u-sw), memory (mem), natural killer (NK), natural killer T (NKT; CD3^+^ CD161^+^ KLRF1^+^), regulatory T (Treg), interferon-stimulated gene (ISG), cytotoxic T lymphocyte (CTL), proliferating (prolif), mucosal-associated invariant T (MAIT), terminally differentiated effector memory CD45RA^+^ T (TEMRA), non-classical (non-class), intermediate (int), classical (class), monocyte (mono), conventional dendritic cell (cDC), plasmacytoid dendritic cell (pDC), migratory (mig), hematopoietic stem and progenitor cell (HSPC).

Having found no evidence to support hypersensitivity myocarditis or antibody-mediated processes, we next sought to take a broad and unbiased approach to uncover immune changes in myopericarditis patients during acute disease. To this end, we first performed serum protein profiling in patients (*n* = 9) and healthy YVC (*n* = 6). Analysis of circulating inflammatory mediators in myopericarditis revealed elevations (false discovery rate (FDR) < 0.05) of the interleukin (IL)-1 family cytokines (IL-1β and IL-1RA) (Fig. 3A), consistent with earlier clinical findings suggesting acute, systemic inflammation. Additionally, the cytokine IL-15, which can activate natural killer (NK) and T cells, was elevated in myopericarditis. Increases in lymphocyte- and monocyte-directed chemokines were also observed (CCL4, CXCL1, and CXCL10) (Fig. 3A). Given the inflammatory profile and cardiac injury observed in myopericarditis patients, we also explored levels of matrix metalloproteases and corresponding counter-regulatory proteins and found they were also elevated (MMP1, MMP8, MMP9, and TIMP1). We next performed exploratory principal component analysis (PCA) involving all measured proteins/cytokines (84 total from Fig. 3A and Fig. S3) to identify whether distinct disease immune phenotypes exist among the patients and compared to healthy YVC. Groups separated well (PC1: 47.44% of variance), indicating clear differences in immune phenotypes and suggesting ongoing cytokinopathy in myopericarditis patients (Fig. 3B and Fig. S4A to D). Notably, patients additionally appeared to separate into two groups (PC2: 21.50% of variance), where one was characterized by MMPs including MMP1 and MMP8 (Fig. 3B and Fig. S4A and B). Upon CMR imaging, patients in this group (P1, P3, P6, and P9) displayed LGE at presentation, whereas the patient in the other group (P2) had no LGE (Table S1).

Complementing our serum analysis, we performed single-cell RNA sequencing (scRNA-seq) on peripheral blood mononuclear cells (PBMCs) of the four patients (P1–4) from whom suitable samples could be collected during acute illness to better define and correlate the immune effectors in vaccine-associated myopericarditis. Since inflammatory effects from vaccination may be transient, we integrated these samples with ones from healthy young male donors matched for time after vaccination (early-young vaccinated controls (E-YVC), *n* = 4). These controls received the third (booster) dose of mRNA vaccine, which has been reported to induce greater inflammatory side effects compared to the second dose (17, 45), thus would enable controlling for the benign inflammation early after vaccination in young adult males and separate the likely pathological signatures in myopericarditis. We additionally included samples from three of the patients at follow-up/recovery (R-P1 191 days, R-P3 195 days, and R-P4 190 days after vaccination), four samples from pediatric male healthy donor (HD) controls, as well as four samples from multisystem inflammatory syndrome in children (MIS-C) after SARS-CoV-2 patients analyzed using cellular indexing of transcriptomes and epitopes by sequencing (CITE-seq (46)) to enable refined annotation of cell subsets using surface protein markers (Fig. 3C, Table S2, and Table S3). After quality control and processing, we obtained a total of 221,727 cells segregating into 44 distinct cell clusters, including unique subsets of myeloid, natural killer (NK), B, and T cells (Fig. 3D and Fig. S5A to D). Notably, we observed the presence of plasma B cells and dividing plasmablasts in myopericarditis patients (Fig. S6A and B), which was further validated using flow cytometry (Fig. S6C). To further evaluate whether these B cells had features of autoantigen-reactive responses, we performed B cell receptor (BCR) repertoire analysis. We observed no significant reduction in clonal diversity or increase in the proportion of IgG clones harboring mutated BCR regions (Fig. S6, D and E), providing little evidence for clonal expansion or somatic hypermutation and supporting findings from our earlier neutralizing antibody and REAP analyses. Differences were observed in the isotype distribution and proportion of cells expressing IgG1 or IgG3 between myopericarditis and healthy E-YVC, though these did not reach statistical significance (Fig. S6, F to I). Overall, these findings potentially suggest non-specific expansion of plasma B cells and plasmablasts due to the broad cytokinopathy and/or secondary responses to systemic inflammation in these patients.

### NK cell dysregulation in myopericarditis

Given the cytokinopathy in myopericarditis and specifically the elevation of IL-15 observed in our serum analysis, we first investigated changes in NK cell subsets. Indeed, the elevation of IL-15 was validated in our scRNA-seq cohort using ELISA (Fig. S7A). Additionally, we noted increased proportions of a CD16^+^ NK cell subset (*p* = 0.087 after correction for multiple comparisons) in patients (Fig. S7B). This trend was not mirrored in innate-like T cells including Vδ2 γδ T and MAIT cells (Fig. S7C and D), possibly indicating unique effects in NK cells. To further investigate whether this NK subset is upregulating genes downstream of the activating cytokine IL-15, we made use of a published gene set of the IL2RB pathway (GSEA Molecular Signatures Database M8615) and saw upregulation of various genes (FDR < 0.05), including both the *IL2RG* and *IL2RB* subunits of the IL-15 receptor (Fig. S7E). To better define the transcriptional signature in this subset, we utilized differential gene expression in comparison to the rest of CD16^+^ NK cells. Top upregulated genes included NK receptors, including the activating receptor NKG2D (*KLRK1*) that recognizes stress-induced cell surface ligands, cytotoxicity-related genes (*GZMA* and *PRF1*), degranulation and activation markers (*LAMP1*, *LAMP2*, and *CD69*), as well as various integrins and chemokines, among others (Fig. S7F). Top downregulated genes notably included various alarmins (*S100A4*, *S100A6*, *S100A10*, and *S100A11*) and interferon-stimulated genes (*ISG15*, *ISG20*, *LY6E*, *IFITM1*, *IFITM3*, and *IFITM2*), which may be associated with subset-specific dysregulated NK cell signaling, as this gene expression pattern was not reflected across other cell types. Similarly, consistent with the known internalization of NKG2D after ligand engagement, which acts as potential negative feedback to desensitize NK cell responses (47, 48), we observed significantly reduced levels of NKG2D protein on the surface of NK cells in patients by flow cytometry (Fig. S7G). One hypothesis is that these parallel analyses of transcription and surface protein potentially reflect recent NK cell cytotoxic engagement, consistent with their capacity to mediate tissue damage. Overall, we describe a signature of NK cell dysregulation and activation, which may be linked to the development of vaccine-associated myopericarditis.

### Expansion of activated cytotoxic T lymphocytes in myopericarditis

**Fig. 4:**
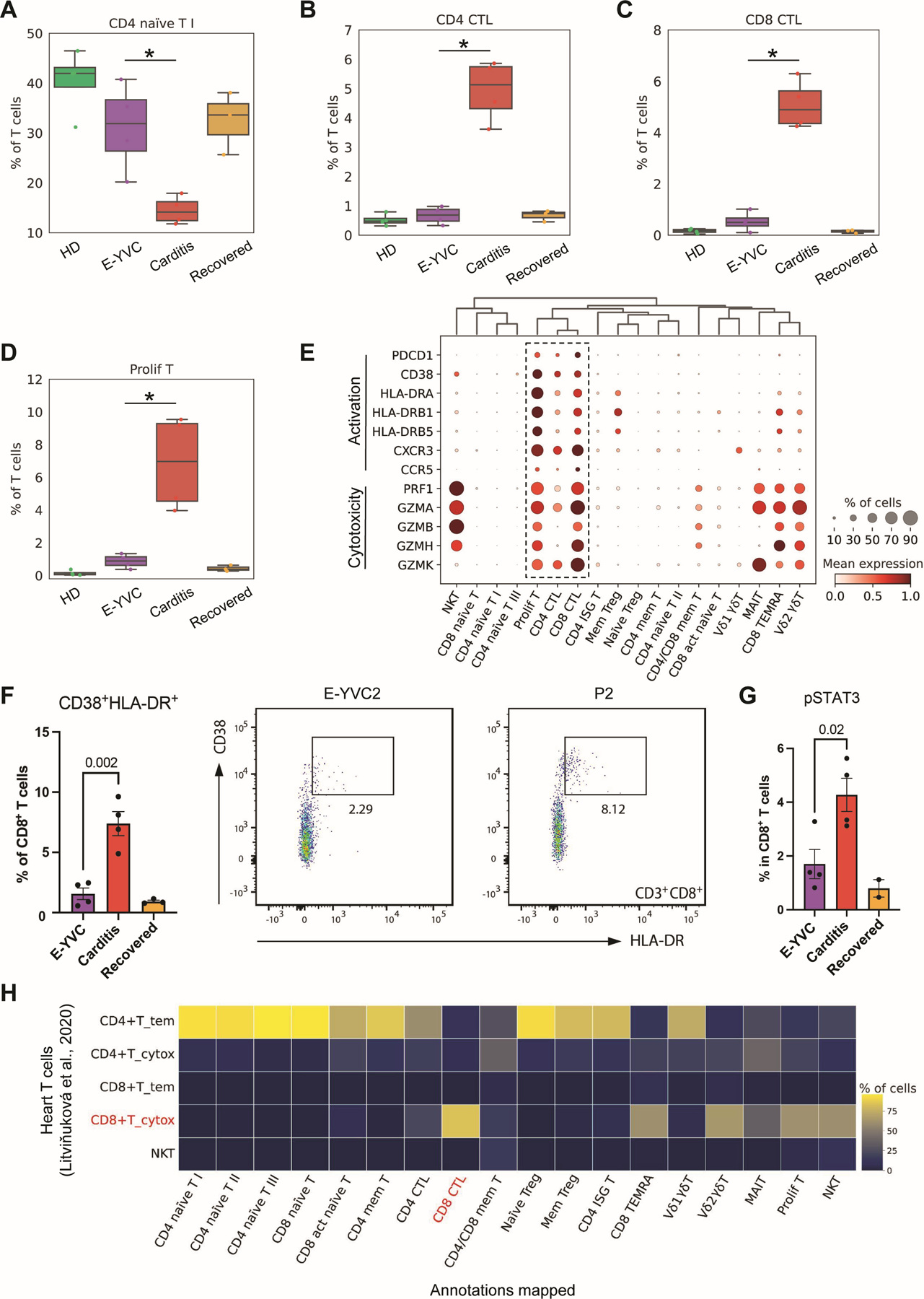
Expansion of activated cytotoxic T lymphocytes in myopericarditis. (**A** to **D**) Box plots showing the average proportions of four T cell subsets (CD4^+^ naïve T I, CD4^+^ and CD8^+^ cytotoxic T lymphocytes (CTLs), as well as proliferating T cells) across the groups. The boxes denote the interquartile range (IQR), horizontal bars represent the median, whiskers extend to 1.5 × IQR, and dots show the values of each donor. Statistical significance was determined using the Bayesian model scCODA (49) accounting for the compositional dependencies between cell subsets in the scRNA-seq data while controlling for false discoveries (FDR < 0.05 in myopericarditis vs. E-YVC). (**E**) Dot plot showing the expression of activation markers (PD-1 and CD38/HLA-DR), chemokine receptors (*CXCR3* and *CCR5*), and cytotoxicity genes (perforin and granzymes) characterizing the T cell subsets shown in (**B** to **D**). (**F**) Flow cytometry quantifying the percentage of the CD38^+^ HLA-DR^+^ population out of CD8^+^ T cells across the groups (left), with representative plots for E-YVC and patient (P2) donors (right). Statistical significance was determined using the unpaired two-tailed *t*-test between the E-YVC and myopericarditis groups, and error bars represent the standard error (SE). (**G**) Percentage phosphorylated STAT3 (pSTAT3) in CD8^+^ T cells across the groups by flow cytometry; statistical significance was determined as in (**F**), and error bars represent SE. (**H**) Heat map depicting the percentage of each T cell subset identified in this study that mapped, using a logistic regression model based on gene expression with mean prediction probability of 0.9, to each subset from published single-cell data of heart T cell populations (60).

Next, to inspect changes in the proportions of T cell subsets across the groups, while accounting for compositional dependencies between the subsets, we employed the Bayesian model scCODA (49) (myopericarditis vs. E-YVC, FDR < 0.05). We observed significantly decreased proportions of a CD4^+^ naïve T cell subset paired with robust expansions of cytotoxic T lymphocytes (CTLs), both CD4^+^ and CD8^+^, as well as proliferating T cells in myopericarditis (Fig. 4A to D). Upon examining the transcriptional signature of these expanded subsets, we noted the unique expression of various activation markers, including PD-1 (*PDCD1*) as well as CD38/HLA-DR (Fig. 4E), which was further validated by flow cytometry in CD8^+^ T cells (Fig. 4F). These CTLs and proliferating T cells further expressed the chemokine receptors CXCR3 and CCR5 (Fig. 4E), whose ligands—CXCL10 and CCL4 respectively—were shown to be significantly elevated in our earlier serum analysis of the patient cohort (Fig. 3A). Notably, these chemokines are known to play central roles in recruitment of T cells to heart tissue, leading to cardiac infiltration of proinflammatory CTLs, and they have previously been described in the context of various inflammatory cardiovascular diseases including classic myocarditis (50–58). Moreover, these cell subsets express perforin (*PRF1*) and a diverse set of granzymes (including *GZMA*, *GZMB*, *GZMH*, and *GZMK*), equipping them to be tissue-damaging (Fig. 4E). Further, CD8^+^ T cells in patients showed significantly increased phosphorylated STAT3 (pSTAT3), indicating ongoing cytokine sensing, compared to E-YVC by flow cytometry (Fig. 4G). In contrast, published studies of healthy vaccinated donors showed maximum elevation in pSTAT3 one day after mRNA secondary vaccination (59). To test the hypothesis that these cells are similar to heart CTLs, we trained a logistic regression model using published single-cell data of heart T cells (60) to predict concordant cell subsets in our dataset based on gene expression. While multiple cell subsets mapped to heart CD4^+^ effector-memory T (CD4^+^ T_tem) cells (suggesting these cells are most transcriptionally similar to blood circulating T cells) and others did not map well to any single heart population (likely due to tissue adaptability or disease-specific signatures), CD8^+^ CTLs mapped with high frequency to the corresponding heart subset (Fig. 4H), potentially suggesting similarities beyond the cytotoxicity profile.

Additionally, to assess the possibility of clonal expansion in these dysregulated T cell subsets, we analyzed the T cell receptor (TCR) repertoire of myopericarditis patients. An assessment of repertoire diversity, as measured by richness and evenness (Shannon index/richness), revealed that non-naïve CD4^+^ and CD8^+^ T cells were highly diverse across cohorts (Fig. S8A). Although CTLs and proliferating T cells were expanded in patients (Fig. 4B to D), we did not see significant changes in the Vβ usage and found little evidence of highly expanded clones in these cells (Fig. S8B and C), though the possibility of autoreactive cells cannot be fully excluded. Of note, myopericarditis patients did not display an expansion of *TRBV11–2*^+^ pathogenic T cells as seen in SARS-CoV-2-associated MIS-C (61–64) (Fig. S8D). In sum, T cells in myopericarditis bear an activated signature despite having comparable diversity to healthy vaccinated controls, consistent with a cytokine-dependent activation of CTLs following vaccination.

### Monocyte dysregulation with evidence of cardiac fibrosis in myopericarditis

**Fig. 5:**
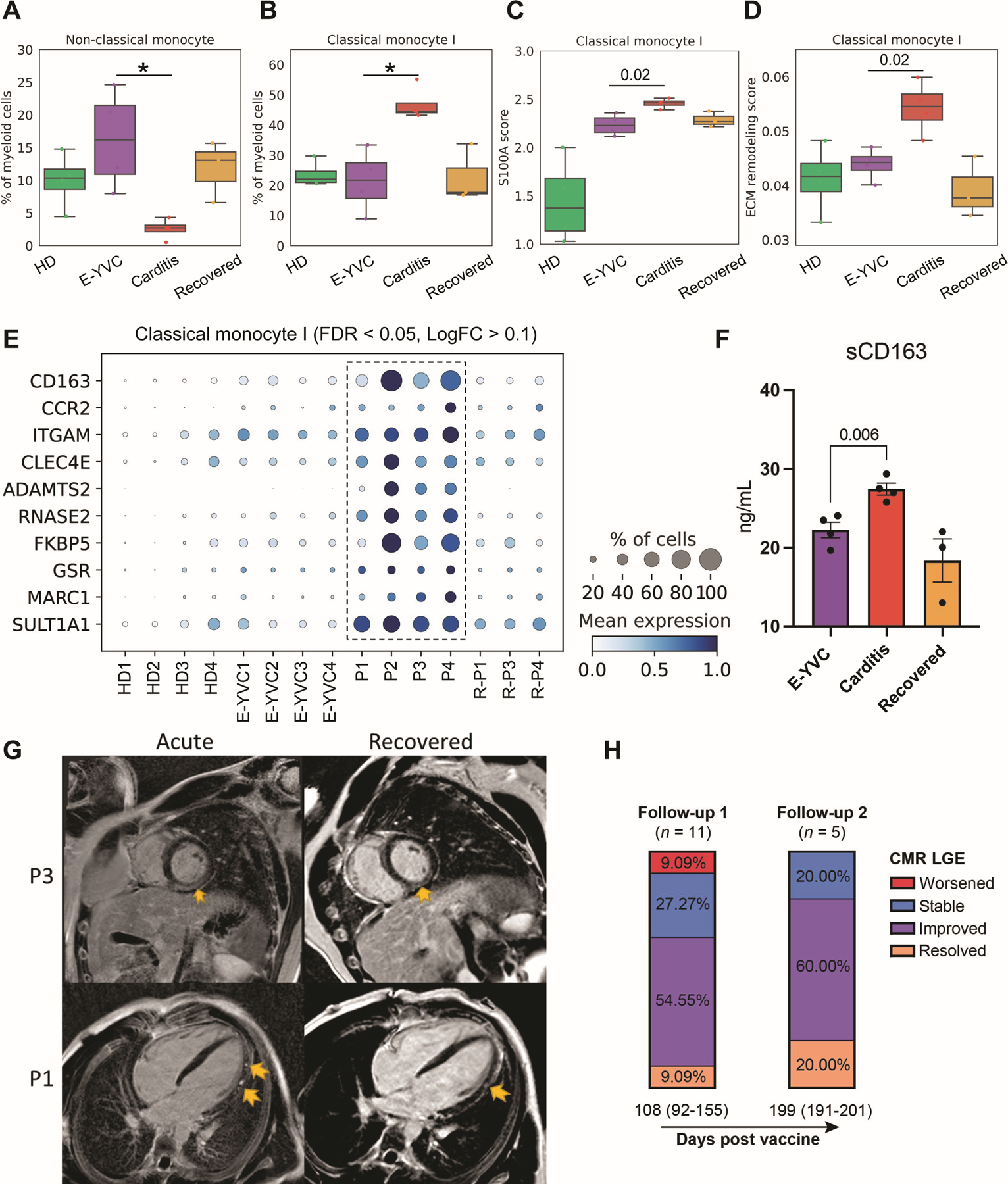
Inflammatory and profibrotic signatures of monocytes in myopericarditis. (**A** and **B**) Box plots showing the average proportions of non-classical (CD14^dim^ CD16^+^) and classical (CD14^+^ CD16^−^) monocyte subsets across the groups. The boxes denote the interquartile range (IQR), horizontal bars represent the median, whiskers extend to 1.5 × IQR, and dots show the values of each donor. Statistical significance was determined using the Bayesian model scCODA (49) accounting for the compositional dependencies between cell subsets in the scRNA-seq data while controlling for false discoveries (FDR < 0.05 in myopericarditis vs. E-YVC). (**C** and **D**) Average expression score of (**C**) inflammatory genes from the S100A family of alarmins (*S100A8–12*; FDR < 0.05, LogFC > 0.1 in myopericarditis vs. E-YVC) and (**D**) 238 genes from a published dataset of extracellular matrix (ECM) remodeling (GSEA Molecular Signatures Database M3468) in the same classical monocyte subset shown in (**B**) across groups. Statistical significance between scores was determined using the unpaired two-sided Wilcoxon rank-sum test comparing the E-YVC and myopericarditis groups. (**E**) Dot plot showing top differentially expressed and upregulated genes in the same classical monocyte subset shown in (**B**) across donors (FDR < 0.05, LogFC > 0.1 in myopericarditis vs. E-YVC). (**F**) Enzyme-linked immunosorbent assay (ELISA) measurement of soluble CD163 (sCD163) in serum across the groups. Statistical significance was determined using the unpaired two-tailed *t*-test between the E-YVC and myopericarditis groups, and error bars represent the standard error (SE). (**G**) Representative cardiac magnetic resonance (CMR) images of acute myopericarditis and follow-up/recovery (191 days for P1 and 82 days for P3 after vaccination) showing persistent late gadolinium enhancement (LGE; yellow arrows) seen in a subset of patients (from 17 patients included in our cohort, at admission, 11 were LGE +, 4 were LGE −, and 2 had no CMR). Particularly, for P1, four chamber phase sequence inversion recovery (PSIR) demonstrating patch sub-epicardial LGE along the left ventricular lateral wall from base to apex (acute), with improvement in both quantity and intensity at follow-up (recovered). For P3, mid ventricle short axis PSIR demonstrating sub-epicardial to nearly transmural LGE sparing the sub-endocardial region (acute), which is mildly improved in intensity and quantity at follow-up (recovered). (**H**) Stacked bar plots depicting the percentage of patients categorized by CMR LGE changes at two follow-ups after vaccination/first admission (median days (IQR)). For exact patients with LGE at admission and follow-up as well as details of imaging findings, see Table S1.

With the extensive inflammatory profile observed in myopericarditis and signs of cardiac injury and LGE on CMR imaging, we lastly investigated changes in the innate myeloid compartment. Compositional analysis (scCODA, FDR < 0.05) revealed a significant decrease in CD14^dim^ CD16^+^ non-classical monocytes, which are commonly anti-inflammatory (65, 66), paired with an increase in inflammatory CD14^+^ CD16^−^ classical monocytes (Fig. 5A and B). These classical monocytes further showed increased expression of genes from the S100A family of alarmins in myopericarditis (Fig. 5C), supporting their role in the inflammatory response and consistent with the elevations we saw in IL-1β (39, 67, 68). Importantly, such classical monocytes can further differentiate into tissue macrophages and contribute to chronic disease (65). To better define this possibility in myopericarditis, we used a published dataset of 238 genes (GSEA Molecular Signatures Database M3468) encoding enzymes/proteins and regulators involved in extracellular matrix remodeling. We observed an upregulation of this remodeling and profibrotic signature in classical monocytes of myopericarditis patients (Fig. 5D), consistent with our earlier serum protein/enzyme analysis in these patients (Fig. 3A). Additionally, we performed differential gene expression analysis (FDR < 0.05, LogFC > 0.1), revealing the upregulation of various specific genes shared across patients (Fig. 5E). Notably, these genes included *CD163*, which marks tissue-resident macrophages (69) and has been associated with a profibrotic phenotype (70). Interestingly, positive CD163 immunostaining was previously reported on myocardial inflammatory infiltrates in myocarditis after COVID-19 (71, 72) and adenovirus-vectored (Ad26.COV2.S) SARS-CoV-2 vaccination (73). These monocytes also expressed *CCR2* (Fig. 5E), which enables migration to sites of tissue injury as well as differentiation into inflammatory cardiac macrophages, including in myocarditis (66, 74–76). Indeed, the abundance of CCR2^+^ macrophages has been associated with cardiac remodeling and fibrosis (74, 77). These results are in agreement with the elevations in various MMPs shown by our earlier serum analysis of the patient cohort (Fig. 3A). Other upregulated genes included ones involved in procollagen processing (*ADAMTS2*), adhesion and migration (*ITGAM*, also known as MAC-1 or CD11b), as well as likely inflammation and oxidative stress (*CLEC4E*, *RNASE2*, *FKBP5*, *GSR*, *MARC1*, *SULT1A1*) (Fig. 5E). Upon monocyte/macrophage activation, CD163 is shed from the cell surface and its serum levels can be further associated with fibrosis (78, 79). In our patients, soluble CD163 (sCD163) was confirmed to be significantly elevated in serum (Fig. 5F). Taken together, our findings potentially provide a mechanistic link to the LGE findings in the majority of patients in our cohort (Fig. 5G), which can persist for months after vaccination (Fig. 5H). These results may further suggest the need for careful long-term monitoring of myocarditis and/or pericarditis patients exhibiting signs of cardiac fibrosis.

In summary, our results point to the effector immune populations in vaccine-associated myopericarditis, consistent with published biopsy reports demonstrating a predominate macrophage and lymphocytic immune infiltrate of affected heart tissue (30, 34–37, 80).

## DISCUSSION

Although rare, vaccine-associated myopericarditis has emerged as a critical area of investigation with important implications for scientists, physicians, and policy makers (81, 82). Understanding how and whether these rare adverse events result from maladaptive immune responses induced by vaccine-vectored antigens, or if they instead result from immune responses triggered by elements of the LNP-mRNA vaccine delivery platform is a question of key importance given the enormous clinical potential for this effective vaccine modality. Furthermore, knowledge about longitudinal clinical outcomes of SARS-CoV-2 mRNA vaccine-associated myopericarditis is currently sparse. In this study, we performed multimodal analyses in a cohort of patients who developed myocarditis and/or pericarditis after receiving an mRNA vaccine to SARS-CoV-2, comprising the first systems-level analysis of the immune landscape in such patients.

In line with prior reports (5, 7, 9, 10, 12, 13), the majority of patients in our cohort were adolescent or young adult males presenting a few days after the second dose of an mRNA vaccine and characterized by acute systemic inflammation, elevated troponin and BNP levels, as well as cardiac imaging abnormalities. First, we show that these patients did not exhibit eosinophilia nor elevated Th2 cytokines, consistent with previous observations (7, 9), making alternative hypotheses of hypersensitivity or eosinophilic myocarditis (11, 33) unlikely explanations of the underlying pathogenesis. Additionally, our analyses of the humoral response to vaccination revealed that myopericarditis patients generated typical or even lower SARS-CoV-2 specific antibodies and neutralizing antibody responses relative to healthy vaccinated controls, consistent with previous results (40). These patients also did not show evidence of cardiac-targeted autoantibodies, which have been reported in SARS-CoV-2 infection (83–85), in agreement with observations reported from one patient (32). While anti-IL-1RA antibodies have been reported in some patients from another report (80), analysis of non-cardiac antigens in a subset of patients from our cohort showed no such autoantibodies (86). Consistently, our BCR repertoire analysis showed little evidence for B cell clonal expansion or somatic hypermutation in patients. These results argue that neither overexuberant nor cross-reacting humoral responses are likely explanations of the pathogenesis (13, 87).

Instead, using an unbiased and systems-based approach, we reveal that vaccine-associated myopericarditis patients are characterized by systemic cytokinopathy and activated cytotoxic lymphocytes with distinct transcriptional signatures consistent with their potential to mediate heart tissue damage. First, we noted an activation signature in NK cells with dysregulation of the activating receptor NKG2D, possibly linked to vaccine-associated myopericarditis. NKG2D is an activating receptor that binds stress ligands on tissues, including the heart (88), inducing cytotoxic effector responses (89–96). Elevation and activation of NK cells were also reported in two vaccine-associated myocarditis/myopericarditis patients from different studies (32, 40). These cellular changes were coupled with elevated serum IL-15, a potent activator of NK and T cells, among other functions (97–100). Next, we show that CD4^+^ and CD8^+^ activated cytotoxic T cells (PD-1^+^ and CD38^+^/HLA-DR^+^) are significantly expanded in myopericarditis early after vaccination, further expressing the chemokine receptors CXCR3 and CCR5 whose ligands, CXCL10 and CCL4 respectively, were concomitantly elevated in patients’ serum. Indeed, as regulators of cytotoxic T cell and Th1 responses, these chemokines play central roles in activated T cell infiltration of cardiac tissue, as demonstrated previously across inflammatory cardiovascular diseases, including classic myocarditis (50–58). Interestingly, using TCR repertoire analysis, we uncovered that these cells did not show evidence of monoclonal expansion, suggestive of an antigen-independent, cytokine-dependent activation following vaccination. T cell activation was noted in a published report from one patient presenting after the first dose of mRNA-1273 vaccination, which centered around IL-18 responses (32), suggesting that such pathology likely overlaps across different clinical presentations and doses. Further, while published mechanistic studies into SARS-CoV-2 vaccine-associated myopericarditis remain scarce, reports from cardiac biopsies support our results, demonstrating lymphocytic infiltration (34–36, 80) including HLA-DR^+^ activated T cells (30). Notably, these findings are distinct from previously reported forms of vaccine-associated myocarditis (including after tetanus toxoid, conjugate meningococcal C and hepatitis B, and smallpox vaccines), where the pathologies were largely eosinophilic (101–103).

Importantly, longitudinal clinical follow-up months after vaccination revealed persistent cardiac imaging abnormalities in some patients, most notably LGE on CMR imaging suggesting cardiac fibrosis (104–106). We additionally observed elevations in various serum extracellular matrix remodeling enzymes (MMP1, MMP8, MMP9, TIMP1), increased inflammatory classical monocytes (CCR2^+^ CD163^+^) carrying a profibrotic signature, and elevation in sCD163, indicative of cardiac macrophage activation. Our clinical, cellular, and molecular findings potentially point to ongoing wound healing, tissue remodeling, and scar formation following cardiac injury in these patients (39, 107–111). Indeed, released damage-associated signals, in addition to elevation of the proinflammatory cytokine IL-1β as seen in patients’ serum, triggered by cardiac injury can induce monocyte/macrophage recruitment, further exacerbating the inflammation in myocarditis and/or resulting in tissue fibrosis (39, 74, 76). These results are supported by published cardiac biopsy reports showing macrophage infiltration of heart tissue (34–36). Most recently, a large clinical study of 69 total patients with clinically suspected SARS-CoV-2 vaccine-associated myocarditis reported 40 biopsy-confirmed cases with prominent T cell and macrophage infiltration of cardiac tissue (80). In one report, overlapping immune infiltrate of T cells and macrophages was additionally found at the vaccine injection site in the deltoid muscle from an autopsy case (112). Another histopathological study of 15 clinically suspected cases further reported cardiac infiltration of HLA-DR^+^ T cells and MAC-1^+^ macrophages (30), which are consistent with the aberrant immune cell subsets we define here. Thus, our molecular and cellular immunological findings herein are supported by published histological evidence, presenting the first characterization of these most likely pathogenic inflammatory cell subpopulations.

The question of why such adverse events develop more frequently after the second dose is intriguing. Recent systems vaccinology approaches revealed that the BNT162b2 mRNA vaccine stimulated only modest innate immune responses after the first dose. These responses were substantially enhanced after secondary immunization (59). The authors further reported a significant increase in plasma IFN-γ and CXCL10 (also known as interferon gamma-induced protein 10) shortly after secondary immunization, proposing a ‘cytokine feedback’ model that regulates innate immune responses. In addition, effective mRNA vaccine responses have also been described to induce systemic IL-15 (113), and other cytokines may be acting locally in the tissue (i.e., not measurably increased in systemic circulation) to potentially drive cytotoxic lymphocyte responses in myopericarditis. IFN-γ further correlated with pSTAT3 levels, which peaked one day after secondary vaccination in healthy donors across several cell types (59). In myopericarditis patients here, pSTAT3 levels in CD8^+^ T cells were elevated several days after vaccination during hospitalization, consistent with sustained cytokinopathy post-vaccination. Indeed, IFN-γ is known to enhance HLA-DR expression on T cells during activation, as observed in our patients, which is further seen in CTL heart infiltration in myocarditis (114). Additionally, CXCR3, the activated T cell homing receptor for IFN-γ-induced CXCL10, has been characterized to facilitate the differentiation of CD8^+^ T cells into short-lived effector, rather than long-lived memory, cells by mediating cell migration based on the strength of the inflammatory stimulus (115–117), which may suggest reduced long-term T cell memory responses to vaccination in such patients with important implications for effective vaccine development. These accumulating pieces of evidence complement our observations, whereby susceptible individuals may experience a heightened cytokine-driven immune response to vaccination, and particularly shortly after the second dose, consequently activating immune effectors and provoking heart inflammation. Whether such responses are governed by virtual memory responses (118) or epigenetic reprogramming of effector subsets and/or innate immune memory (119–122) is a fundamental question warranting future investigation.

While the LNP component of the vaccine alone was found to be highly inflammatory, such responses centered on IL-6 and IL-1β (41, 123). IL-1β was elevated in our cohort of patients, and together with upstream NLRP3 inflammasome activation and associated cytokines may play a role in the pathogenesis of myocarditis (32, 39). However, IL-1β induction by lipid-formulated RNA vaccines, which can then stimulate various proinflammatory cytokines, was also shown to be dependent on both the RNA and lipid formulation in human immune cells (124). Thus, a compound role of the adjuvant delivery platform in synergy with vaccine-vectored antigens is more likely the driver of an exaggerated immune cytokine response driving cardiac pathology following vaccination in susceptible individuals. What causes certain individuals, notably adolescent and young adult males, to be more susceptible to these cardiac-related adverse events is not clear but likely not unique to vaccine-induced pathogenesis. A bias toward younger males is similarly seen in community-acquired myocarditis/pericarditis, where many large-scale epidemiological and clinical studies have demonstrated that patients are much more frequently males (65–84% of patients) and significantly younger than female patients (125–132). The male sex and young age have also been described as risk factors for stronger cardiac inflammation and a higher incidence of fibrosis in myocarditis (133, 134). Future studies defining sex and age differences in immune responses to mRNA vaccination, as well as cardiac biopsy data, are needed to elucidate these mechanisms further. Analysis of data from COVID-19 mRNA vaccine clinical trials shows both higher efficacy in males compared to females as well as more systemic adverse events (e.g., fever, fatigue, and headache) in younger individuals (1, 3, 135, 136), potentially suggesting heightened immune and/or inflammatory responses in these demographic groups.

Our study has some limitations. While our cohort of LNP-mRNA vaccine-associated myopericarditis is one of the largest studied to date, and our hypothesis is consistent with published reports from other patients, the number of subjects remains limited to make broad conclusions. Additionally, despite using several control groups to ensure our findings are unique to vaccine-associated myopericarditis, variations in age, vaccine dose, or time after vaccination across individuals are considerations for interpretation and broad conclusions. Our high-throughput autoantibody analysis by REAP also focused on extracellular/secreted antigens, so intracellular autoantigens may still play a role in the pathogenesis, though this was not supported in previous studies (32). Lastly, our study did not have tissue samples to confirm the effector immune population infiltrating the heart, yet our results agree with previously published biopsy reports.

Based on our findings, longitudinal clinical monitoring of vaccine-associated myopericarditis patients may be warranted, given the possibility of persistent cardiac abnormalities. It is also critical to contextualize the risk of adverse events and potential clinical sequelae following SARS-CoV-2 vaccination (2, 5) compared to the risk of sequelae, hospitalization, and/or death from complications following infection with SARS-CoV-2 (5, 24, 137–140). Our study leverages a rare patient cohort and presents findings with potential implications for vaccine development and clinical care. Future studies building on the translational relevance of our work will be important to further optimize the excellent safety profile of mRNA vaccines among specific demographic sub-groups.

In conclusion, our findings likely rule out some previously proposed mechanisms of mRNA vaccine-associated myopericarditis and implicate aberrant cytokine-driven lymphocyte activation and cytotoxicity as well as inflammatory and profibrotic monocyte responses in the immunopathology occurring in susceptible patients following mRNA vaccination.

## MATERIALS AND METHODS

### Study design

The primary aim of this study was to investigate the systemic immunopathological landscape underlying rare cases of myopericarditis occurring after SARS-CoV-2 LNP-mRNA vaccination. A total cohort of 23 patients developing myocarditis and/or pericarditis with elevated troponin, BNP, and CRP levels as well as cardiac imaging abnormalities shortly after vaccination was compared to healthy vaccinated controls among other groups. Different subsets of these patients were profiled using multimodal approaches, including SARS-CoV-2 antibodies and neutralization, high-throughput exoproteome cardiac autoantibody profiling, serum proteomics, flow cytometry, ELISA, as well as peripheral blood single-cell transcriptome and repertoire sequencing to define immunopathological alterations. Lastly, clinical follow-up including CMR imaging was conducted between 2 to 9 months after vaccination to assess potential long-term disease effects.

### Human subjects research

Human subjects in this study provided informed consent to use their samples for research and to publish de-identified data, in accordance with Helsinki principles for enrollment in research protocols that were approved by the Institutional Review Board (IRB) of Yale University (protocols 2000028924 and 1605017838). For a subset of patients, whose inclusion was solely for medical record review for population surveillance and vaccine investigations, the IRB approved the study and waived the requirement for informed consent (protocol 2000028093). All relevant ethical regulations for work with human participants were followed.

### Blood sample processing

For the antibody, neutralization, REAP, and cytokine assays, whole blood was collected in heparinized CPT blood vacutainers (BDAM362780, BD) and processed on the same day of collection. Plasma samples were collected after centrifugation of whole blood at 600*g* for 20 minutes at room temperature. Undiluted plasma was transferred to 1.8 mL Eppendorf polypropylene tubes and stored at −80 °C for subsequent analysis. For blood clinical parameters, significance was determined through comparison against normal reference ranges provided by the CLIA-certified Yale New Haven Hospital Department of Laboratory Medicine for each test.

For the scRNA-seq analysis, PBMCs were first isolated by Ficoll-Paque PLUS (GE Healthcare) or Lymphoprep (STEMCELL Technologies) density gradient centrifugation. Cells were subsequently washed twice in phosphate-buffered saline (PBS) and resuspended in complete RPMI 1640 (cRPMI) medium (Lonza) containing 10% fetal bovine serum (FBS), 2 mM glutamine, and 100 U/ml each of penicillin and streptomycin (Invitrogen). PBMCs were then stored at 10^6^ cells/ml in 10% dimethyl sulfoxide (DMSO) in FBS and stored in −80°C overnight before further storage in liquid nitrogen. Serum was isolated by centrifugation of serum tubes and saving the supernatant in aliquots, which were flash frozen in liquid nitrogen prior to cryopreservation in −80°C. All patient PBMCs were processed and cryopreserved within 1–6 hours of blood draw. Healthy donor PBMCs were all processed within 24 hours, to account for overnight shipping.

### SARS-CoV-2 specific antibodies

ELISA was performed as previously described (141). In short, Triton X-100 and RNase A were added to serum samples at final concentrations of 0.5% and 0.5  mg/ml, respectively, and incubated at room temperature for 30 minutes before use to reduce risk from any potential virus in serum. MaxiSorp plates (96 wells; 442404, Thermo Scientific) were coated with 50 μL per well of recombinant SARS-CoV-2 S total (SPN-C52H9–100 μg, ACROBiosystems), S1 (S1N-C52H3–100 μg, ACROBiosystems), and RBD (SPD-C52H3–100 μg, ACROBiosystems) at a concentration of 2 μg/mL in PBS and were incubated overnight at 4 °C. The coating buffer was removed, and plates were incubated for 1 hour at room temperature with 200 μL of blocking solution (PBS with 0.1% Tween-20 and 3% milk powder). Plasma was diluted serially at 1:100, 1:200, 1:400, and 1:800 in dilution solution (PBS with 0.1% Tween-20 and 1% milk powder), and 100 μL of diluted serum was added for 2 hours at room temperature. Human anti-spike (SARS-CoV-2 human anti-spike clone AM006415; 91351, Active Motif) and anti-nucleocapsid (SARS-CoV-2 human anti-nucleocapsid clone 1A6; MA5–35941, Invitrogen) were serially diluted to generate a standard curve. Plates were washed three times with PBS-T (PBS with 0.1% Tween-20) and 50 μL of HRP anti-human IgG antibody (1:5,000; A00166, GenScript) diluted in dilution solution was added to each well. After 1 hour of incubation at room temperature, plates were washed six times with PBS-T. Plates were developed with 100 μL of TMB Substrate Reagent Set (555214, BD Biosciences) and the reaction was stopped after 5 minutes by the addition of 2 N sulfuric acid. Plates were then read at wavelengths of 450 nm and 570 nm.

### Cell lines and virus

Vero E6 kidney epithelial cells (C1008, CRL-1586) were cultured in Dulbecco’s Modified Eagle Medium (DMEM) supplemented with 1% sodium pyruvate, non-essential amino acids and 5% FBS at 37 °C and 5% CO_2_. The cell line was obtained from the American Type Culture Collection (ATCC) and was negative for contamination with mycoplasma using commercially available kits. SARS-CoV-2 (ancestral strain, D614G) USA-WA1/2020 was obtained from BEI Resources (NR-52281) and minimally amplified in Vero E6 cells to generate working stocks of virus. All experiments were performed in a Biosafety Level 3 facility with approval from the Yale Environmental Health and Safety office.

### Neutralization assay

Patient and vaccinated control sera were isolated as above, and heat treated for 30 minutes at 56 °C. Plasma was serially diluted from 1:10 to 1:2430 and incubated with SARS-CoV-2 (ancestral strain, D614G) for 1 hour at 37 °C. Inoculum was subsequently incubated with Vero E6 cells in a six-well plate for 1 hour for adsorption. Next, infected cells were overlayed with DMEM supplemented with NaHCO_3_, 2% FBS and 0.6% Avicel mixture. Plaques were resolved at 40 hours after infection by fixing in 4% formaldehyde for 1 hour followed by staining in 0.5% crystal violet. All experiments were performed in parallel with negative control sera with an established viral concentration sufficient to generate 60–120 plaques in control wells.

### REAP

Antibody purification, yeast adsorption and library selections, NGS library preparation, and REAP score calculation were performed as previously described (43, 44, 86). A more detailed protocol is also included in the Supplementary Methods.

### Cytokine analysis

Plasma was isolated from patients as described above. Levels of cytokines were assessed using commercially available vendors, described at length previously (142). Briefly, sera were shipped to Eve Technologies (Calgary, Alberta, Canada) on dry ice, and levels of cytokines and chemokines were measured using the Human Cytokine Array/Chemokine Array 71–403 Plex Panel (HD71) and the Human Matrix Metalloprotease Panel (HDMYO-3–12). All samples were measured upon first thaw. Samples below the range of detection were replaced with the lowest observed value for quantitation.

Principal component analysis and hierarchical clustering were performed using MATLAB 2020b (MathWorks, Inc.) using standard builtin functions. Gene enrichment was performed on identified clusters using GO analysis to identify significance, and the top 10 hierarchical, biological processes were reported (143, 144).

### ELISA

Serum was isolated as above alongside PBMC isolation, flash frozen, and stored at −80⁰C until further use. Serum was then thawed at 37⁰C and plated in duplicate or triplicate. Standards were made fresh for each assay. The assays were run using the ELISA MAX Deluxe Set Human IL-15 (BioLegend, Cat 435104) and the CD163 Human ELISA (ThermoFisher, Cat. EHCD163) kits following the manufacturer’s instructions.

### Single-cell RNA-sequencing and alignment

Cryopreserved PBMCs were thawed in a water bath at 37 °C for ~2 minutes and removed from the water bath when a tiny ice crystal remained. Cells were transferred to a 15 mL conical tube of pre-warmed growth medium. The cryovial was rinsed with additional growth medium (10% FBS in DMEM) to recover leftover cells, and the rinse medium was added to the 15 mL conical tube while gently shaking the tube.

Thawed PBMCs were centrifuged at 400*g* for 8 minutes at RT, and the supernatant was removed without disrupting the cell pellet. The pellet was resuspended in 1X PBS with 2% FBS, and cells were filtered with a 30 μM cell strainer. The cellular concentration was adjusted to 1,000 cells/μL based on the cell count and cells were immediately loaded onto the 10x Chromium Next GEM Chip G, according to the manufacturer’s user guide (Chromium Next GEM SingleCell V(D)J Reagent Kits v1.1). We aimed to obtain a yield of ~10,000 cells per lane.

cDNA libraries for gene expression and TCR/BCR sequencing were generated according to the manufacturer’s instructions (Chromium Next GEM SingleCell V(D)J Reagent Kits v1.1). Each library was then sequenced on an Illumina NovaSeq 6000 platform. The single-cell transcriptome sequencing data were processed using CellRanger v5.0.1 and aligned to the GRCh38 reference genome (145). CITE-Seq surface protein reads were quantified using a provided tagged antibodies dictionary.

### Quality control, preprocessing, and integration

After data alignment and quantification, we performed quality control assessment before proceeding with data processing and downstream analysis. First, genes expressed in fewer than five cells, as well as cells with fewer than 200 genes or more than 10% mitochondrial gene fraction were removed.

The filtered single-cell data were thereafter integrated and batch effects corrected using single-cell variational inference (scVI) (146) with a generative model of 64 latent variables and 500 iterations. More specifically, scVI’s negative binomial model was employed on raw counts, selecting 5,000 highly variable genes identified by the tool’s native method in the combined datasets to produce latent variables. To minimize the dependence of clustering on cell cycle effects, previously defined cell cycle phase-specific genes in the Seurat package (147) were excluded from this list of highly variable genes. Data from acute myopericarditis patients, E-YVC, recovered patients, and HD were integrated for downstream analysis. Four MIS-C CITE-Seq samples were also included to aid with cell annotation using surface protein markers.

The latent representation generated by scVI was used to compute the neighborhood graph (*scanpy.pp.neighbors*), which was utilized for Louvain clustering (*scanpy.tl.louvain*) as well as Uniform Manifold Approximation and Projection (UMAP) visualization (*scanpy.tl.umap*).

Before clustering, to aid with cell annotation, Single-Cell Remover of Doublets (Scrublet) (148) was used to preliminary label doublets by computing a doublet score for each cell. In particular, a Student’s *t*-test (*p* < 0.01) and Bonferroni correction were used within fine grained sub-clustering of each cluster initially identified by the Louvain algorithm.

Data processing was performed using the Scanpy toolkit (149) following published recommended standard practices. Before downstream analysis, data were normalized (*scanpy.pp.normalize_per_cell*, scaling factor = 10^4^) and log-transformed (*scanpy.pp.log1p*). For heat maps of gene expression, the data were further scaled (*scanpy.pp.scale*, max value = 10).

### Cluster identification and immune subset annotation

First, a logistic regression model was used to transfer preliminary cell annotations from our previous published MIS-C dataset (63) using highly variable genes (*scanpy.pp.highly_variable_genes*) shared between the two datasets to guide cluster identification. Thereafter, clustering was performed using the Louvain algorithm with an initial resolution of three, and the resulting clusters were defined based on the expression of published cell-specific gene markers. For ambiguous clusters, differential gene expression analysis compared to all other clusters was performed (*scanpy.tl.rank_genes_groups*), and the resulting top genes were used for unbiased determination of cell identity.

The initial doublet predications by Scrublet were revised here and doublets confirmed based on cluster co-expression of heterogeneous lineage gene markers (e.g., *CD3*, *CD19*, *CD14*) along with the n_counts and n_genes distributions. In addition to removing doublets, clusters displaying patterns of dying cells with high mitochondrial content were further removed. Refined subclusters were used for downstream data analysis and visualization, including cell type frequency, differential gene expression, and repertoire analyses.

### Cell subset proportions

To determine significant changes in the proportions of identified subsets across the groups, while accounting for compositional dependencies between the subsets in the scRNA-seq data, we employed the Bayesian model scCODA (49). Specifically, for each subset, the myopericarditis group was compared to the E-YVC group, and default parameters were used. Changes classified by scCODA as credible after correction for multiple comparisons (FDR < 0.05) were considered statistically significant. In some cases where changes were not classified as significant by scCODA’s method, we employed the non-parametric unpaired two-sided Wilcoxon rank-sum test with Benjamini-Hochberg FDR correction for multiple comparisons as previously described (150) and we report the exact adjusted *p*-value, given that such changes may still be biologically meaningful.

### Differential gene expression

The limma package (151) was used to obtain differentially expressed genes (FDR < 0.05). The analysis was performed either comparing different cell subsets or in the same cell subset comparing cells from different groups. Top differentially expressed genes by logFC (upregulated and downregulated) as well as published pathway gene sets were used for plotting and heat map visualization after data scaling.

### Gene expression scores

Published gene sets from the Molecular Signatures Database (mSigDB) were used to calculate the average enrichment scores across cells of defined subsets as specified in the figure legends using the *scanpy.tl.score_genes* function with default parameters.

### Mapping annotations between datasets

For mapping single-cell immune populations between datasets, a logistic regression model was employed using scikit-learn with default parameters. For heart cells, T lymphocyte subsets from published single-cell data (60) were used to train the model using expression data of highly variable genes (*scanpy.pp.highly_variable_genes*) shared between the two datasets. The model was then applied to predict matching cell subsets in our dataset (mean prediction probability = 0.9), and the percentage of cells mapping between subsets is visualized.

### TCR repertoire analysis

TCR diversity, gene usage, and clonotype analyses were done as previously described (63). A more detailed protocol is also included in the Supplementary Methods.

### BCR repertoire analysis

Analysis of the BCR repertoire was performed as previously described (63) using the Immcantation framework as listed above in the TCR analysis and annotations incorporated from overall PBMC clustering for B cell subsets.

### Flow cytometry

Patient and donor PBMCs were thawed as above and rested in cRPMI for 2 hours at 37°C before staining. The cells were counted, resuspended in PBS, and stained with e780 Fixable Viability Dye (eBioscience, Cat. 65–0865-14) for 15 minutes on ice. The cells were then washed and resuspended in PBS with 10% FBS, with Human TruStain FcX (BioLegend, Cat. 422302) and True-Stain Monocyte Blocker (BioLegend, Cat. 426102) for 15 minutes on ice. The cells were then stained with surface antibody cocktails for 25 minutes on ice. For phospho-staining, cells were first treated with Fc blocker and then fixed and permeabilized with methanol before staining for intracellular proteins. All flow cytometry data was acquired on the LSR Fortessa cytometer, and gating strategies are shown in Fig. S9 and Fig. S10. Further details about flow cytometry antibodies are included in the Supplementary Methods.

### Statistical analysis

Data were analyzed by various statistical tests as described in detail in the corresponding individual figure captions. For all comparisons, error bars, exact *p*-values, and testing levels were shown whenever possible and corrections for multiple comparisons were done when appropriate.

## Supplementary Material

MDAR checklist

table S3

data file S1

main supplementary material
