# Supplementary material for: Cytokinopathy with aberrant cytotoxic lymphocytes and pro-fibrotic myeloid response in SARS-CoV-2 mRNA vaccine-associated myocarditis
Source: Sci Immunol. Author manuscript; Available in PMC 2024 May 12. (PMC10468758; doi:10.1126/sciimmunol.adh3455)
Supplement: main supplementary material [file NIHMS1914947-supplement-main_supplementary_material.docx]

Cytokinopathy with aberrant cytotoxic lymphocytes and pro-fibrotic myeloid response in SARS-CoV-2 mRNA vaccine-associated myocarditis

**Authors:** Anis Barmada^1,^*, Jon Klein^1,^*, Anjali Ramaswamy^1,‡^, Nina N. Brodsky^1,2,‡^, Jillian R. Jaycox^1^, Hassan Sheikha^1,2^, Kate M. Jones^1^, Victoria Habet^2^, Melissa Campbell^2^, Tomokazu S. Sumida^3^, Amy Kontorovich^4^, Dusan Bogunovic^4,5^, Carlos R. Oliveira^2^, Jeremy Steele^2^, E. Kevin Hall^2^, Mario Pena-Hernandez^1^, Valter Monteiro^1^, Carolina Lucas^1,10^, Aaron M. Ring^1^, Saad B. Omer^6,7,8^, Akiko Iwasaki^1,9,10,#^, Inci Yildirim^2,7,8,10,#^, Carrie L. Lucas^1,#^

**SUPPLEMENTARY MATERIALS**

Supplementary Methods: REAP, TCR repertoire analysis, & flow cytometry antibodies

Tables S1 to S3 (Table S3 is a separate Excel file)

Figures S1 to S10

Data file S1. Raw data file (Excel spreadsheet)

MDAR Reproducibility Checklist

**Supplementary Methods:**

*REAP*

REAP was performed as previously validated and described in detail (43, 44, 86). More specifically, for antibody purification, Triton X-100 and RNase were added to patient plasma at a final concentration of 0.5% and 0.5 mg/ml, respectively, and incubated for 30 minutes to inactivate enveloped RNA viruses. 20 µL protein G magnetic resin (Lytic Solutions) was washed and resuspended in PBS, then added to 50 µL of inactivated plasma. Serum-resin mixture was incubated for three hours at 4 ⁰C with shaking. Resin was washed with PBS and resuspended in 90 µL 100 mM glycine pH 2.7 for 5 minutes. Supernatant was extracted and added to 10 µL sterile 1M Tris pH 8.0. For yeast adsorption, empty vector (pDD003) yeast were induced by culture in 1:10 SDO-Ura:SGO-Ura for 24 hours. 10^8^ induced yeast were washed with PBE (PBS with 0.5% BSA and 0.5 mM EDTA), resuspended with 100 µL purified IgG, and incubated for three hours at 4 ⁰C with shaking. Yeast-depleted IgG was eluted from the Yeast-IgG mixture through 0.45 µm filter plates by centrifugation at 3000*g* for 3 minutes.

For yeast library selections, the Exo201 or 204 yeast library was induced at an OD of 1 cultured in 1:10 SDO-Ura:SGO-Ura at 30⁰C. Prior to selection, 5^8^ induced yeast were set aside to allow for comparison of the pre-selection to post-selection libraries. 10^8^ induced yeast were washed with PBE and added to wells of a sterile 96-well plate. 10 µg of yeast adsorbed IgG were added to the yeast library in duplicate in 100 µL PBE and incubated for 1 hour at 4⁰C. Yeast were washed PBE and incubated with 1:100 biotin anti-human IgG Fc antibody (clone HP6017, BioLegend, or clone QA19A42, BioLegend) for 30 minutes. Yeast were washed with PBE and incubated with a 1:20 dilution of Streptavidin MicroBeads (Miltenyi Biotec) for 30 minutes. Yeast were resuspended in PBE and IgG-bound yeast were isolated by positive magnetic selection using the MultiMACS M96 Separator (Miltenyi Biotec) according to manufacturer instructions and as previously described (43, 44). Selected yeast were resuspended in 1 mL SDO-Ura at 30 ⁰C for 24 hours.

For NGS library preparation, DNA was extracted from yeast libraries using Zymoprep-96 Yeast Plasmid Miniprep kits or Zymoprep Yeast Plasmid Miniprep II kits (Zymo Research) according to standard manufacturer protocols. A first round of PCR was used to amplify a DNA sequence containing the protein display barcode on the yeast plasmid, as previously described (43, 44). A second round of PCR was performed on 1 µL step 1 PCR product using Nextera i5 and i7 dual-index library primers (Illumina), as previously described (43, 44). PCR products were pooled, run on a 1% agarose gel, and DNA corresponding to the band at 257 base pairs was cut. DNA (NGS library) was extracted using a QIAquick Gel Extraction Kit (Qiagen) according to standard manufacturer protocols. NGS library was sequenced using an Illumina NextSeq550 and an NextSeq high output sequencing kit with 75 base pair single-end sequencing according to standard manufacturer protocols. A minimum of 200,000 reads on average per sample was collected and the pre-selection library was sampled at ten times greater depth than other samples. Samples with fewer than 50,000 reads were discarded as failed sequencing.

For REAP score calculation, barcode counts were extracted from raw NGS data using custom codes and counts from technical replicates were summed. Next, aggregate and clonal enrichment was calculated using edgeR (152) and custom codes. Aggregate enrichment is the log2 fold change of all barcodes associated with a particular protein summed in the post-library relative to the pre-library, with zeroes in the place of negative fold changes. Log2 fold change values for clonal enrichment were calculated in an identical manner, but barcode counts across all unique barcodes associated with a given protein were not summed. Clonal enrichment for a given reactivity was defined as the fraction of clones out of total clones that were enriched (log2 fold change ≥ 2). Aggregate ([E_a_](https://www.codecogs.com/eqnedit.php?latex=E_a#0)) and clonal enrichment ([E_c_](https://www.codecogs.com/eqnedit.php?latex=E_c#0)) for a given protein, a scaling factor ([β_u_](https://www.codecogs.com/eqnedit.php?latex=%5Cbeta_u#0)) based on the number of unique yeast clones (yeast that have a unique DNA barcode) displaying a given protein, and a scaling factor ([β_f_](https://www.codecogs.com/eqnedit.php?latex=%5Cbeta_f#0)) based on the overall frequency of yeast in the library displaying a given protein were used as inputs to calculate the REAP score, which is defined as follows.

REAP score = [E_a_](https://www.codecogs.com/eqnedit.php?latex=E_a#0) × ([E_c_](https://www.codecogs.com/eqnedit.php?latex=E_c#0))^2^ × [β_u_](https://www.codecogs.com/eqnedit.php?latex=%5Cbeta_u#0) × [β_f_](https://www.codecogs.com/eqnedit.php?latex=%5Cbeta_f#0)

[β_u_](https://www.codecogs.com/eqnedit.php?latex=%5Cbeta_u#0) and [β_f_](https://www.codecogs.com/eqnedit.php?latex=%5Cbeta_f#0) are logarithmic scaling factors that progressively penalize the REAP score of proteins with low numbers of unique barcodes or low frequencies in the library, and have been described in detail previously (43, 44). Antigens with an average REAP score greater than 0.5 across all samples were defined as “sticky” and excluded from further analysis. Autoantibody reactivities were defined as antigens with REAP score > 2 and > 1.96 row- (antigen-) wise Z score threshold.

*TCR repertoire analysis*

*Diversity analysis*

Raw sequencing reads were processed by the CellRanger 10x V(D)J pipeline and assembled into V(D)J contigs across cells. High confidence V(D)J contigs were retained for downstream analysis. First, we aligned contigs to the IGMT reference database using the Change-O V1.0.0 pipeline (153). Second, we filtered cells by removing those with ambiguous beta or alpha chain, or no beta chain. We retained cells with more than one of each chain, assigning unique chains with the highest nUMI. To calculate diversity across annotated cell populations, we computed repertoire richness and Shannon diversity using the Alakazam package (v1.2.0) (153). To account for sequencing depth and cell type proportion differences between donors, donor cells were first randomly down-sampled to the smallest number of cells in per annotation group. To assess diversity, cells were then randomly down-sampled for 200 iterations, and the mean metric across these iterations was reported.

*Gene usage & clonotype analysis*

Gene usage was quantified using the scRepertoire (v1.3.5) package, and TCR contigs were preprocessed as above (154). V-gene and J-gene distribution was visualized for non-naïve CD8^+^ and CD4 ^+^ proliferating/cytotoxic populations. PCA was performed to visualize the V- and J-gene distributions. Clonotypes were defined as cells with identical alpha and beta chains, comprising VDJC genes as well as the CDR3 region.

*Flow cytometry antibodies*

The antibodies used were: Anti-CD19 (BioLegend, Cat. 302234, Lot. B296493), Anti-CD8 (BD, Cat. 563256, Lot. 1203279), Anti-CD4 (BioLegend, Cat. 300536, Lot. B332220), Anti-NKG2D (BioLegend, Cat. 320819, Lot. B324671), Anti-CD38 (BioLegend, Cat. 303522, Lot. B316154), Anti-HLA-DR (BioLegend, Cat. 307610, Lot. 278650), Anti-CD27 (BioLegend, Cat. 356412, Lot. B324801), Anti-CD3 (BioLegend, Cat. 300424, Lot. B347088), Anti-pStat3 (BioLegend, Cat. 651009, Lot. B339294), Anti-CD56 (BioLegend, Cat. 362506, Lot. B2863371), and Anti-CD16 (BioLegend, Cat. 302026, Lot. B266048).

**
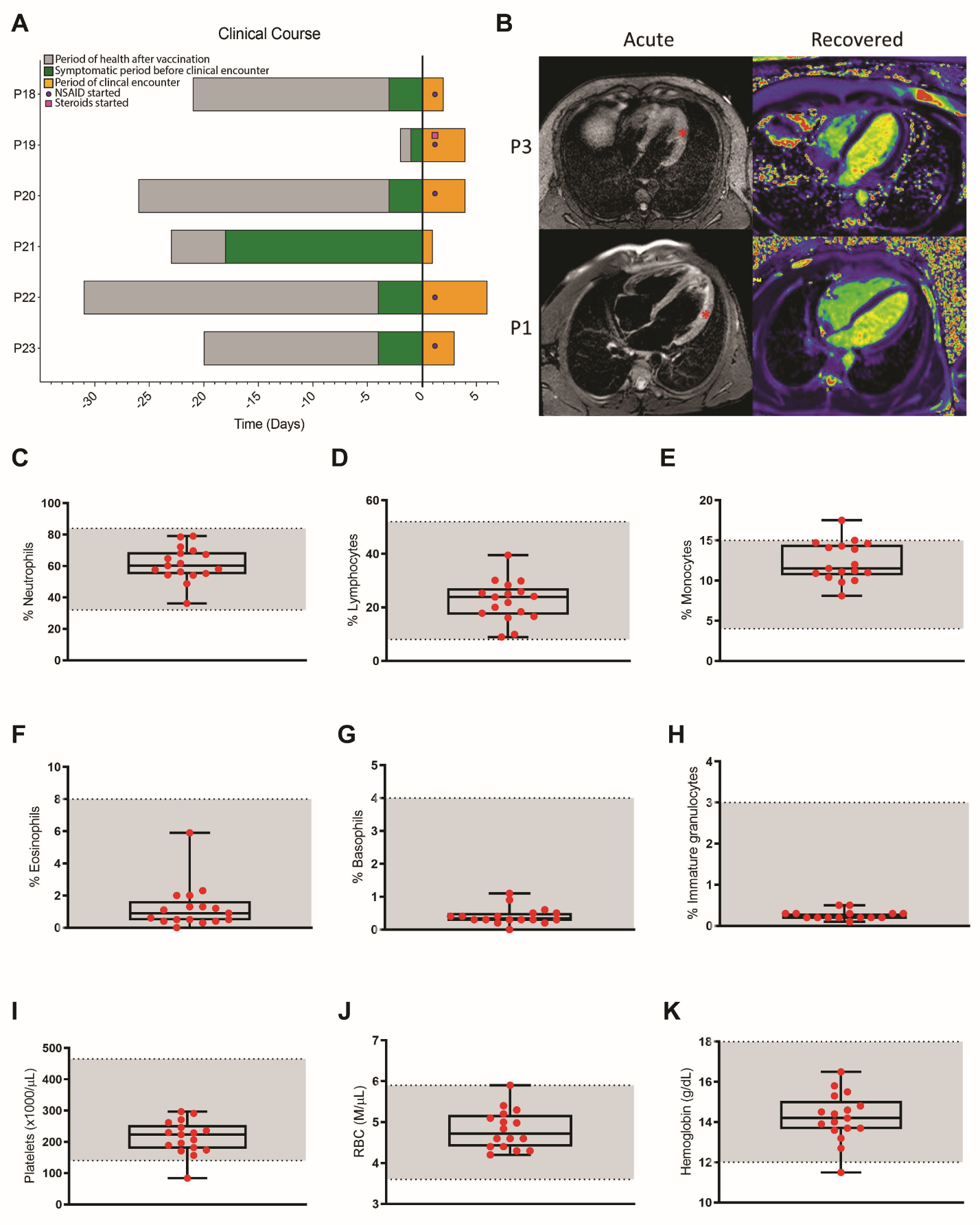
**

**Fig. S1: Additional clinical information and complete blood count differential for the myopericarditis cohort.** (**A**) Time course for excluded patients showing the day of vaccine administration, symptom onset, and treatment relative to hospital admission (Day 0). These six patients either first experienced symptoms after a delay of > 7 days after vaccination (P18, P20, P22, and P23) or were (incidentally) positive for SARS-CoV-2 by polymerase chain reaction (PCR) testing upon hospital admission (P19 and P21), and thus were excluded from subsequent analyses. (**B**) Representative cardiac magnetic resonance (CMR) images of acute myopericarditis and follow-up/recovery showing the resolution of edema. For both patients, four chamber T2 turbo spin echo sequence with fat saturation demonstrating increased signal intensity of the myocardium in a ratio greater than 1.9 > 1 compared to skeletal muscle (acute), and four chamber T2 parametric map demonstrating uniform relaxation time along the entire left ventricular myocardium at a relaxation rate of less than 55 msec indicating no residual edema (recovered). (**C** to **K**) Blood values in patients tested during hospital admission. Boxes depict the interquartile range (IQR), horizontal bars represent the median, whiskers extend to 1.5 × IQR, and red dots show the value of each patient. Dashed lines and gray area represent normal reference ranges as provided by the CLIA-certified Yale New Haven Hospital Department of Laboratory Medicine. Abbreviations: red blood cells (RBC).


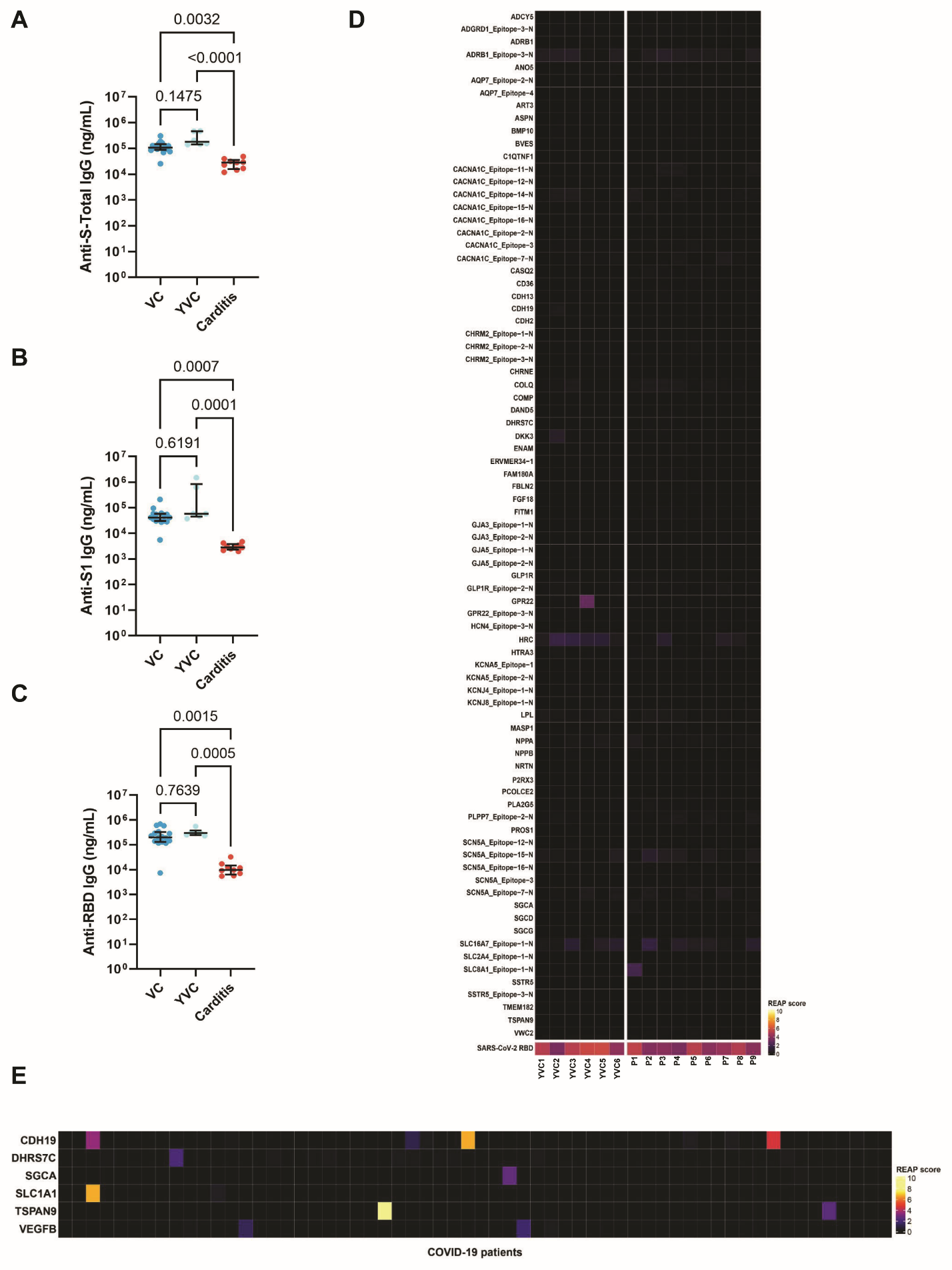


**Fig. S2: SARS-CoV-2-specific antibodies and absence of cardiac autoantibodies in myopericarditis.** (**A** to **C**) Levels of SARS-CoV-2 specific antibodies among patients (*n* = 9) compared to healthy vaccinated controls (VC, *n* = 16), in addition to those closer in age to patients where there is an increased risk for vaccine-associated myopericarditis (younger vaccinated controls (YVC), *n* = 6). Black bars denote the group median and error bars represent the interquartile range (IQR). Statistical significance was assessed using the Kruskal-Wallis test with Dunn's correction for multiple comparisons. (**D**) Heat map of Rapid Extracellular Antigen Profiling (REAP) scores showing all cardiac autoantibodies in the library as defined by the Human Protein Atlas (heart tissue enriched/enhanced, 81 total) regardless of reactivity across individual patients (*n* = 9) and YVC (*n* = 6). SARS-CoV-2 RBD antigen is used as a positive control in vaccinated individuals. (**E**) REAP heat map showing positive cardiac-related autoantibodies detected in several individuals from a cohort of hospitalized COVID-19 patients (*n* = 60).


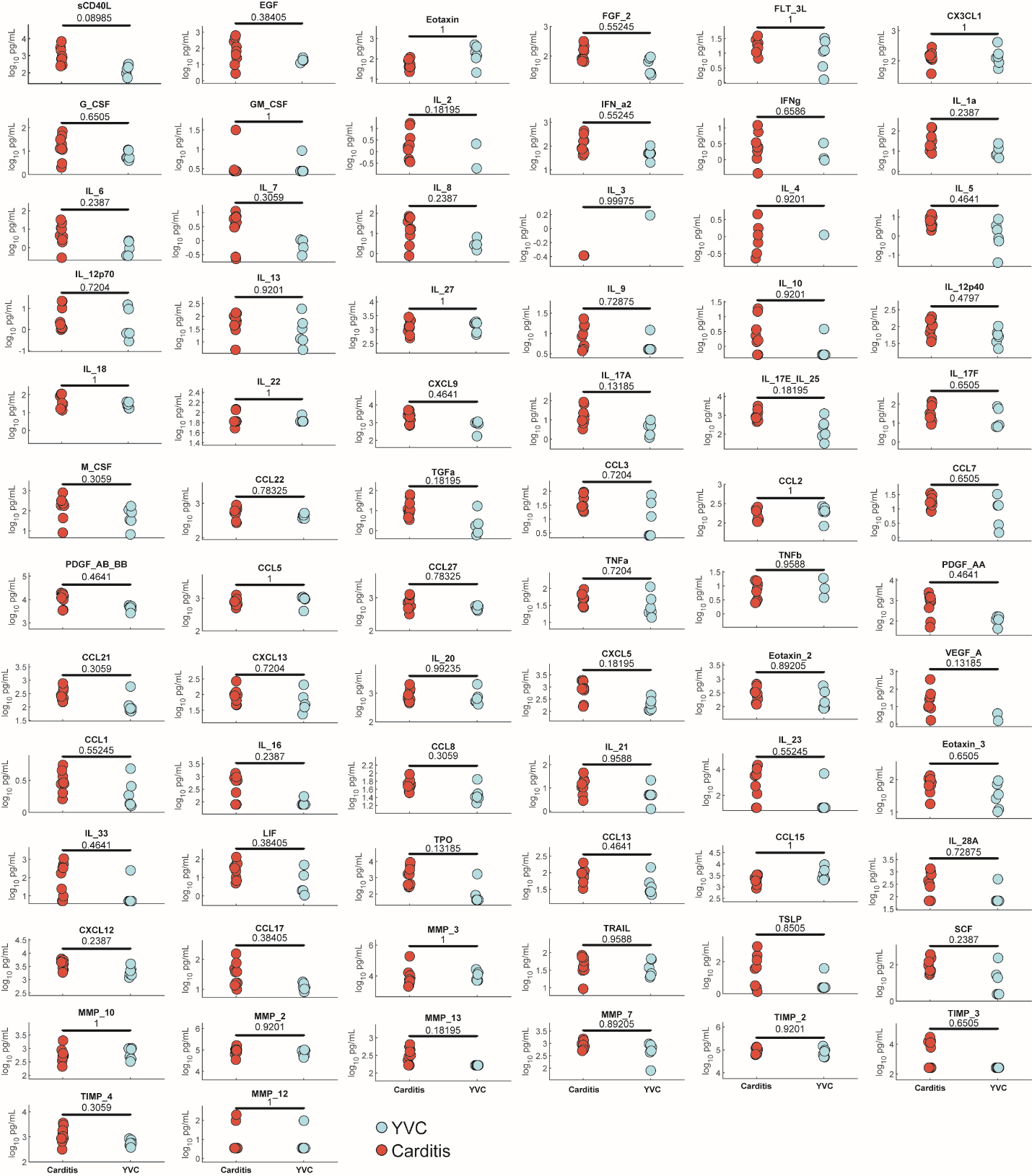


**Fig. S3: Additional serum proteins from myopericarditis patients.** Scatter plots of all additional serum proteins and cytokines assayed from patients (*n* = 9) and healthy younger vaccinated controls (YVC, *n* = 6). Initial exact *p*-values were calculated using max-T procedure for permutation testing.


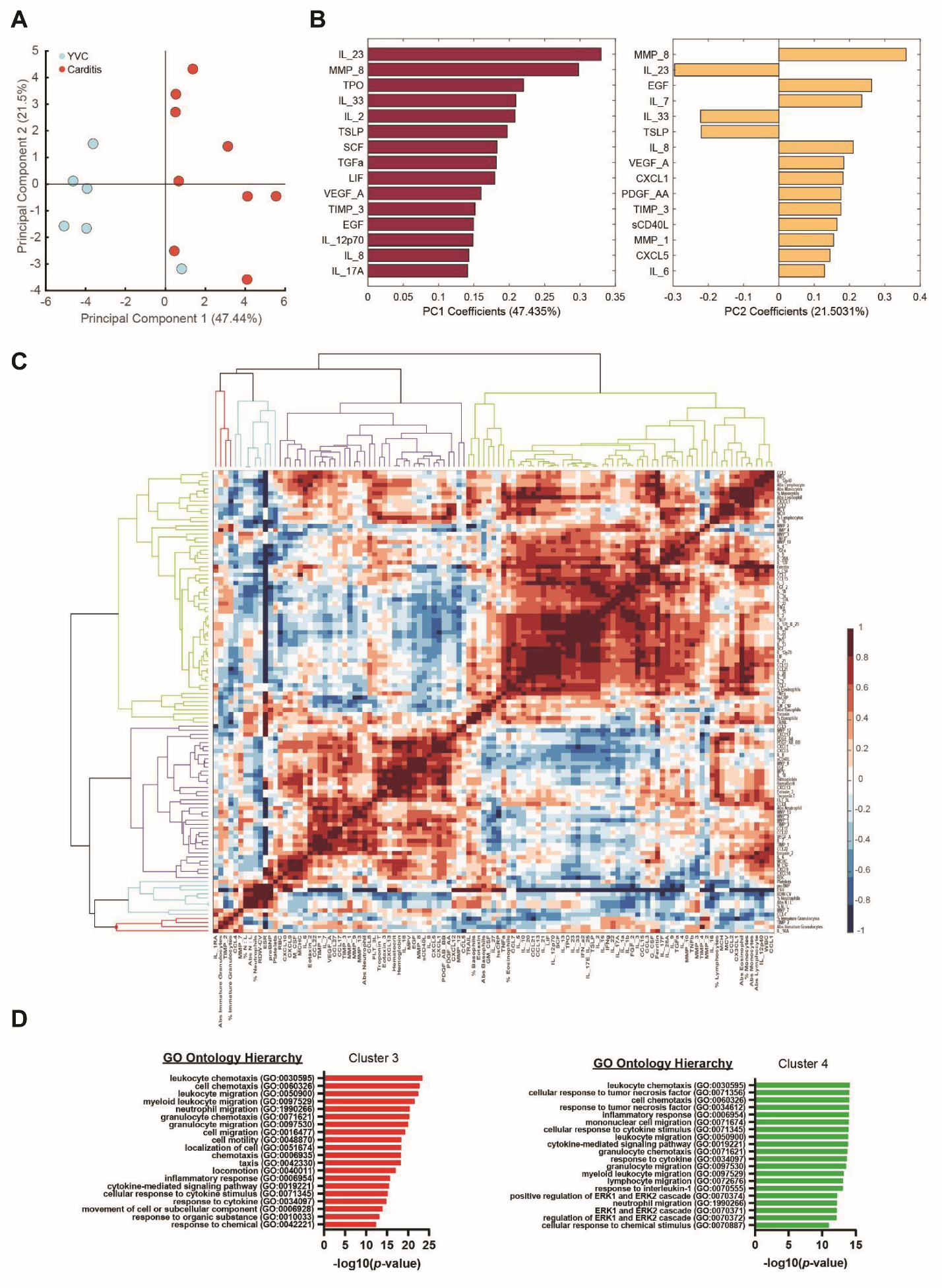


**Fig. S4: Serum cytokine dysregulation in myopericarditis.** (**A**) Principal component analysis (PCA) of all serum proteins assayed (84 total) between patients (*n* = 9) and healthy younger vaccinated controls (YVC, *n* = 6), and (**B**) loadings for PC1 and PC2 (related to **Fig. 3B**). (**C**) Heat map showing the four clusters of covarying cytokines and clinical parameters among patients. (**D**) Gene Ontology (GO) term enrichment analysis of the two largest clusters (3 and 4) shown in (**C**).

**
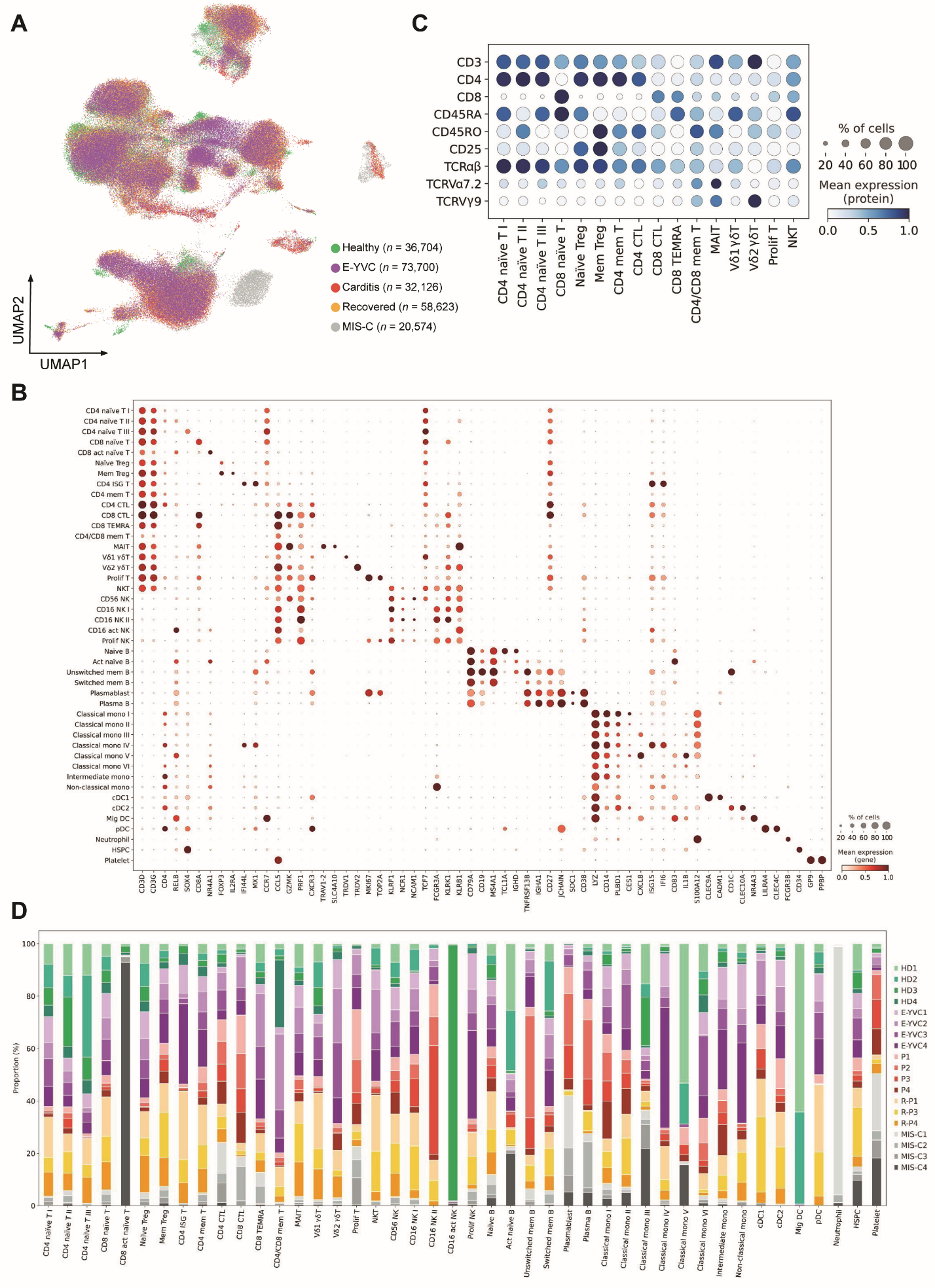
**

**Fig. S5: Single-cell RNA-seq analysis approach.** (**A**) UMAP visualization (221,727 cells) of the integrated and filtered dataset separated by condition, including pediatric male healthy donors (*n* = 4), healthy early-young vaccinated controls (E-YVC, *n* = 4), acute myopericarditis patients (*n* = 4), and recovered patients at follow-up (*n* = 3), as well as CITE-seq multisystem inflammatory syndrome in children (MIS-C) after SARS-CoV-2 samples (*n* = 4) included to refine cluster annotation using surface protein markers (source of the majority of neutrophils in the dataset). (**B** and **C**) Dot plots showing standardized mean expression of selected cell-specific marker genes (**B**) and surface proteins (**C**) defining the immune cell populations identified in **Fig. 3D**. (**D**) Stacked bar plots depicting the proportion of cells across identified subsets classified by donor.


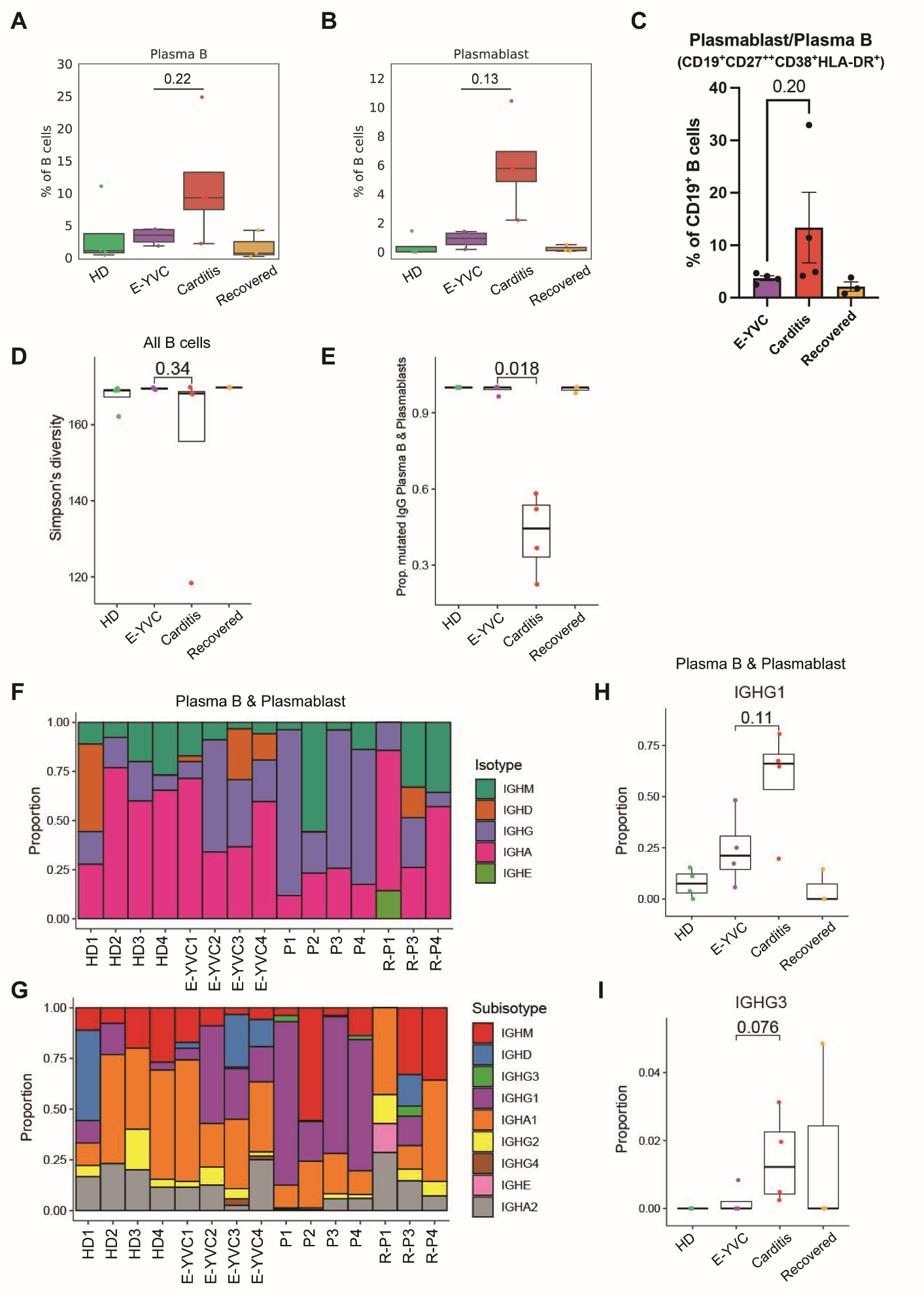


**Fig. S6: B cell subset and receptor repertoire analysis.** (**A** and **B**) Box plots depicting average proportions of plasma B and plasmablasts (dividing) from scRNA-seq between groups. Boxes denote the interquartile range (IQR), horizontal bars represent the median, whiskers extend to 1.5 × IQR, and dots show the values of each donor. Statistical significance was determined using the unpaired two-sided Wilcoxon rank-sum test with Benjamini-Hochberg FDR correction for multiple comparisons, and the exact adjusted *p*-value is reported. (**C**) Percentage of circulating plasmablasts/plasma B cells (CD19^+^ CD27^++^ CD38^+^ HLA-DR^+^) within CD19^+^ PBMCs by flow cytometry quantified across groups. Statistical significance was computed using the unpaired two-tailed *t*-test between the E-YVC and myopericarditis groups, and error bars represent the standard error (SE). (**D** and **E**) Box plots showing Simpson’s diversity computed in total B cells across groups (**D**), and proportion of mutated IgG clones in combined plasma B and plasmablasts across groups (**E**) from the single-cell data. Statistical significance was determined using the unpaired two-sided Wilcoxon rank-sum test. (**F** and **G**) Distribution of isotypes (**F**) and sub-isotypes (**G**) across donors in combined plasma B and plasmablasts. (**H** and **I**) Isotype frequencies of *IGHG1* (**H**) and *IGHG3* (**I**) within combined plasma B and plasmablasts quantified across groups. Statistical significance was determined using the unpaired two-sided Wilcoxon rank-sum test.

**
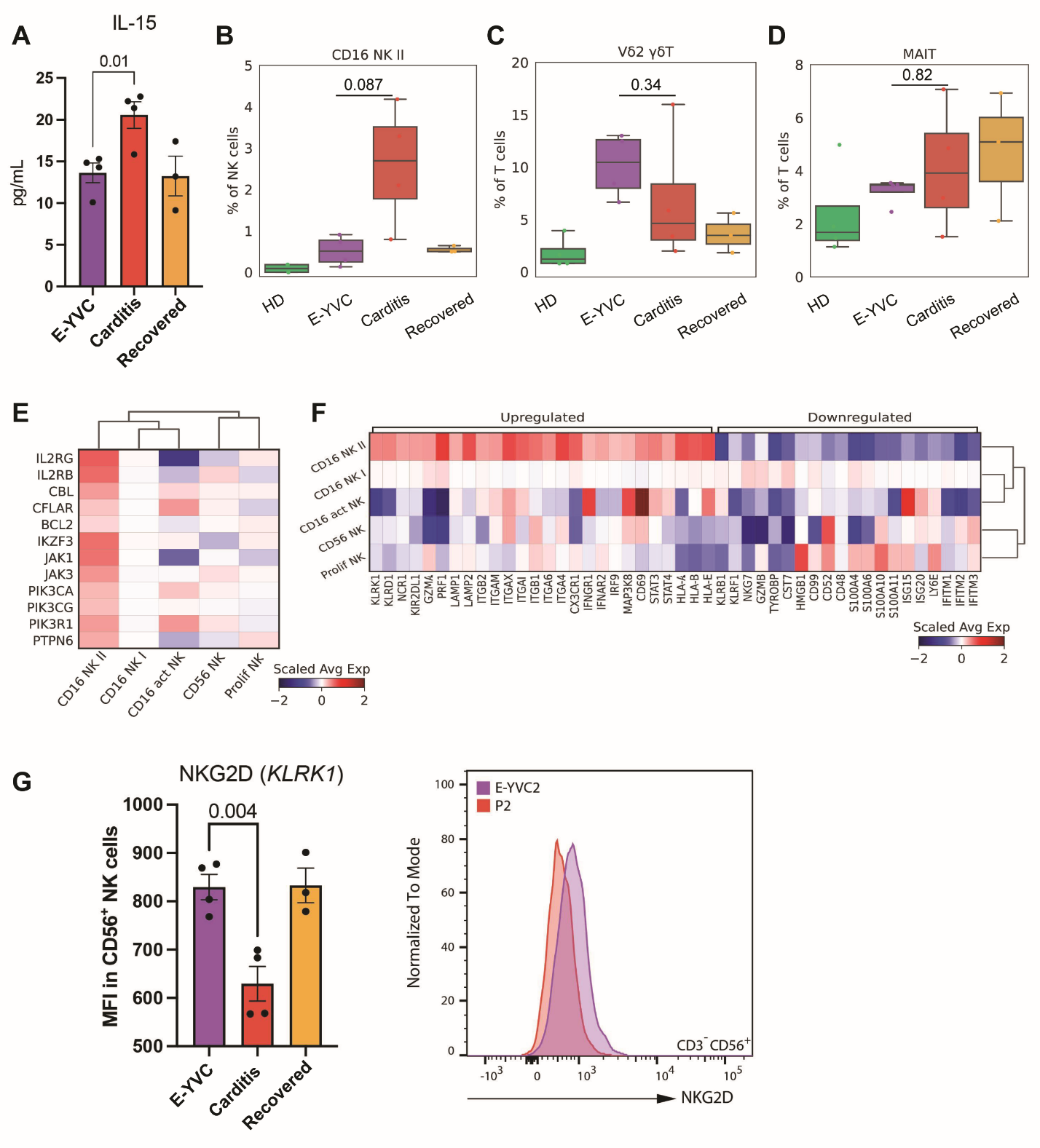
**

**Fig. S7: NK cell dysregulation and activation signature in myopericarditis.** (**A**) Enzyme-linked immunosorbent assay (ELISA) measurement of IL-15 across the groups. Statistical significance was determined using the unpaired two-tailed *t*-test between the myopericarditis and E-YVC groups, and error bars represent the standard error (SE). (**B** to **D**) Box plots depicting the average proportion of the (**B**) CD16^+^ NK II, (**C**) Vδ2 γδ T, and (**D**) MAIT subsets between groups. Boxes denote the interquartile range (IQR), horizontal bars represent the median, whiskers extend to 1.5 × IQR, and dots show the values of each donor. Statistical significance was determined using the unpaired two-sided Wilcoxon rank-sum test with Benjamini-Hochberg FDR correction for multiple comparisons, and the exact adjusted *p*-value is reported (myopericarditis vs. E-YVC). (**E** and **F**) Heat maps depicting the scaled average expression across NK subsets of differentially expressed genes in the CD16^+^ NK II subset compared to the rest of CD16^+^ NK cells (FDR < 0.05), either selected from the (**E**) BioCarta IL2RB pathway (M8615) genes, or from the (**F**) top upregulated and downregulated genes. (**G**) Flow cytometry analysis showing the mean fluorescent intensity (MFI) of NKG2D in CD3^−^ CD56^+^ NK cells (both CD16^+^ and CD16^−^) across the groups (left), with representative histograms for E-YVC and patient (P2) donors (right). Statistical significance was determined as in (**A**), and error bars represent SE.


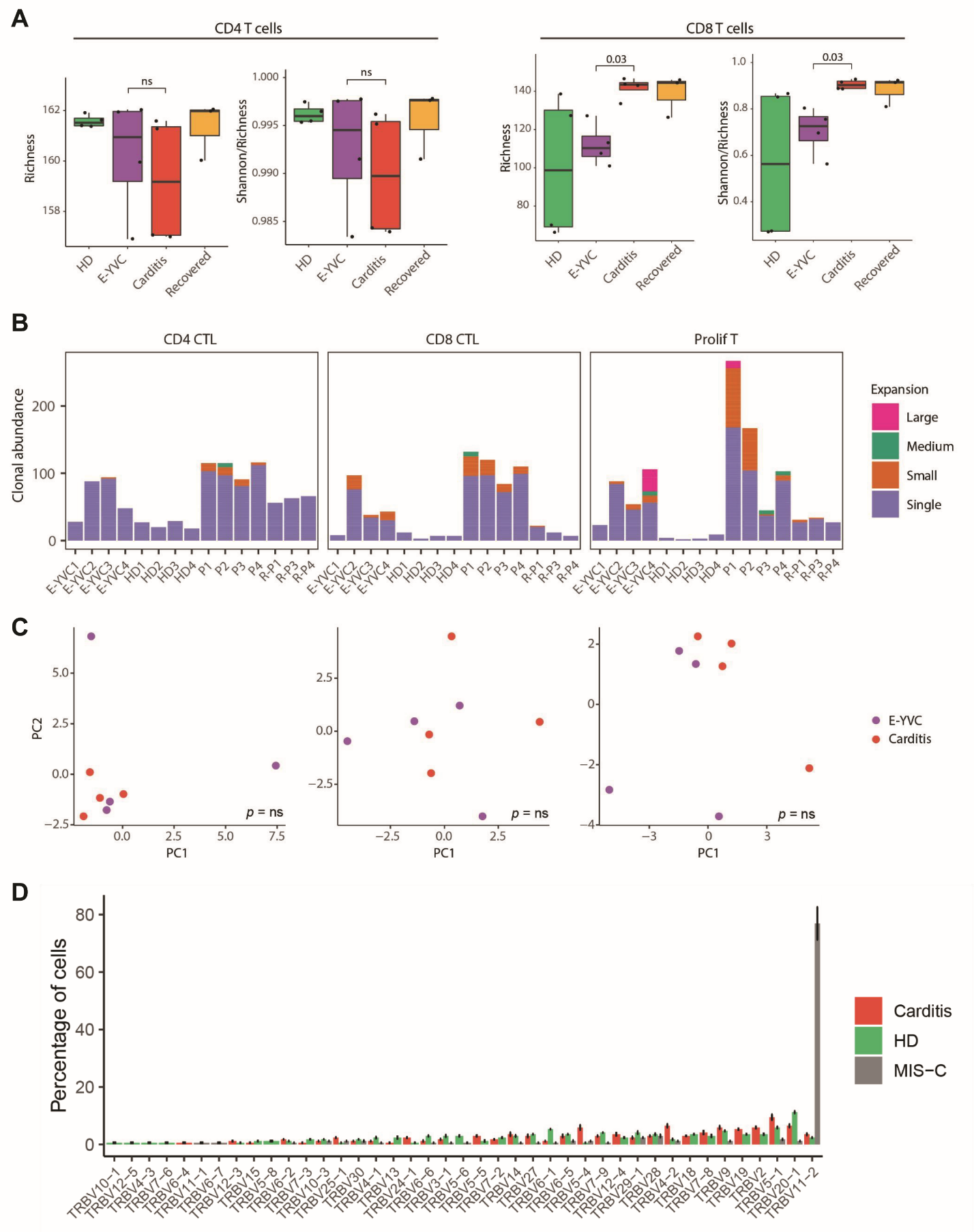


**Fig. S8: Single-cell T cell receptor repertoire analysis shows little evidence for monoclonal expansion in myopericarditis.** (**A**) TCR diversity quantified by Shannon index/richness and richness in non-naïve CD4^+^ and CD8^+^ T cells across groups. (**B**) Distribution of clonotype abundances across donors in cytotoxic T lymphocytes (CTLs), both CD4^+^ and CD8^+^, as well as proliferating T cell clusters. Clonal expansions are binned into categories (Single = 1 clone; Small, 0 < n < 5; Medium, 5 < n < 10; Large, n > 10). (**C**) Principal component analysis (PCA) of donor V-gene frequency within the cell subsets shown in (**B**) for myopericarditis vs. healthy early-young vaccinated controls (E-YVC). Cells were down-sampled to the lowest cell number per condition. Statistical significance was calculated by permutation test, where the statistic refers to the ratio of mean intra/inter cluster Euclidean distances among points. (**D**) *TRBV* gene distribution in CD4^+^ and CD8^+^ CTLs as well as proliferating T cell clusters comparing myopericarditis patients with SARS-CoV-2-associated multisystem inflammatory syndrome in children (MIS-C) patients and healthy donors (HD).


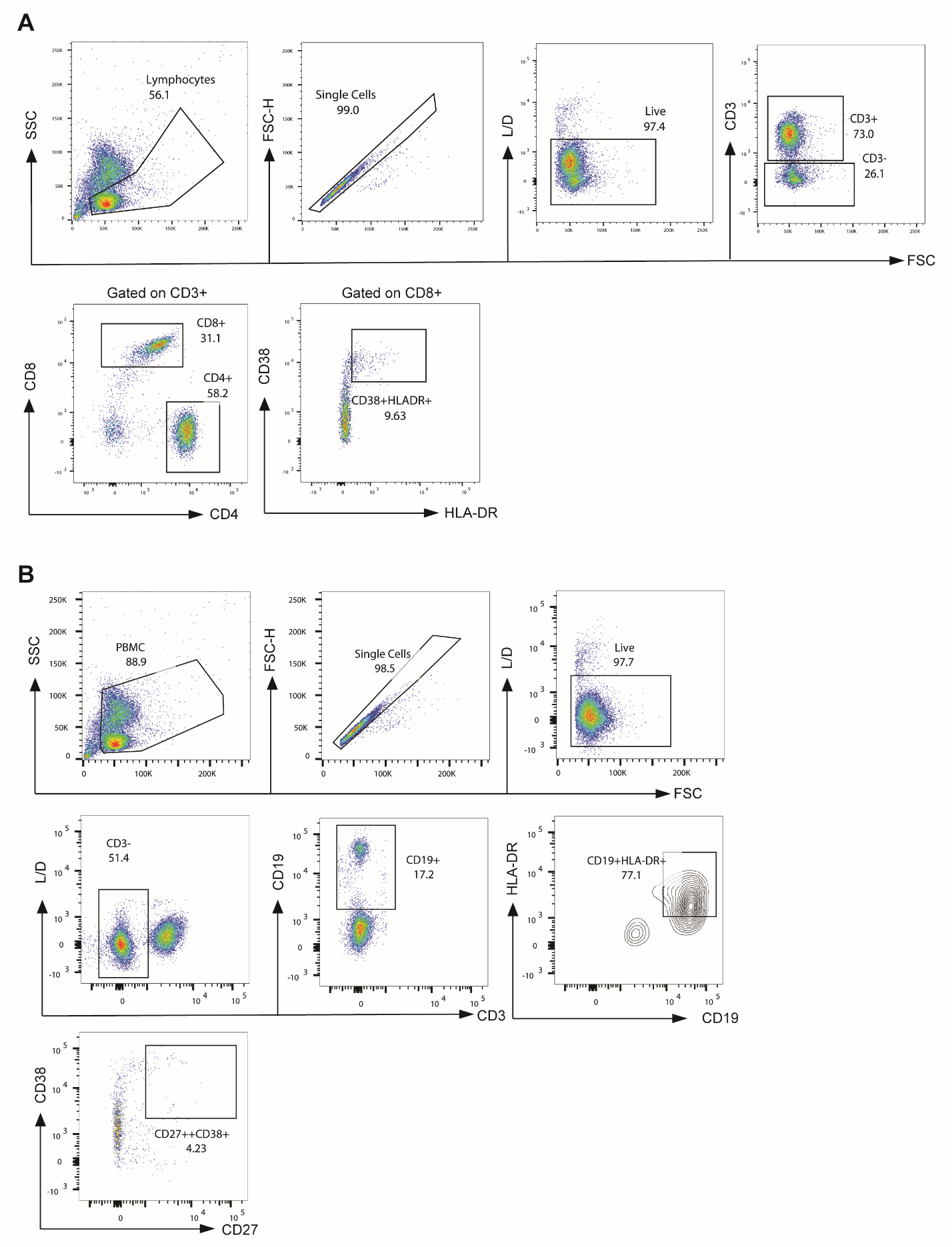


**Fig. S9**: **T cell and plasmablast flow cytometry gating strategies.** Gates are shown for (**A**) CD38^+^HLA-DR^+^ CD8^+^ T cells and (**B**) plasmablasts.


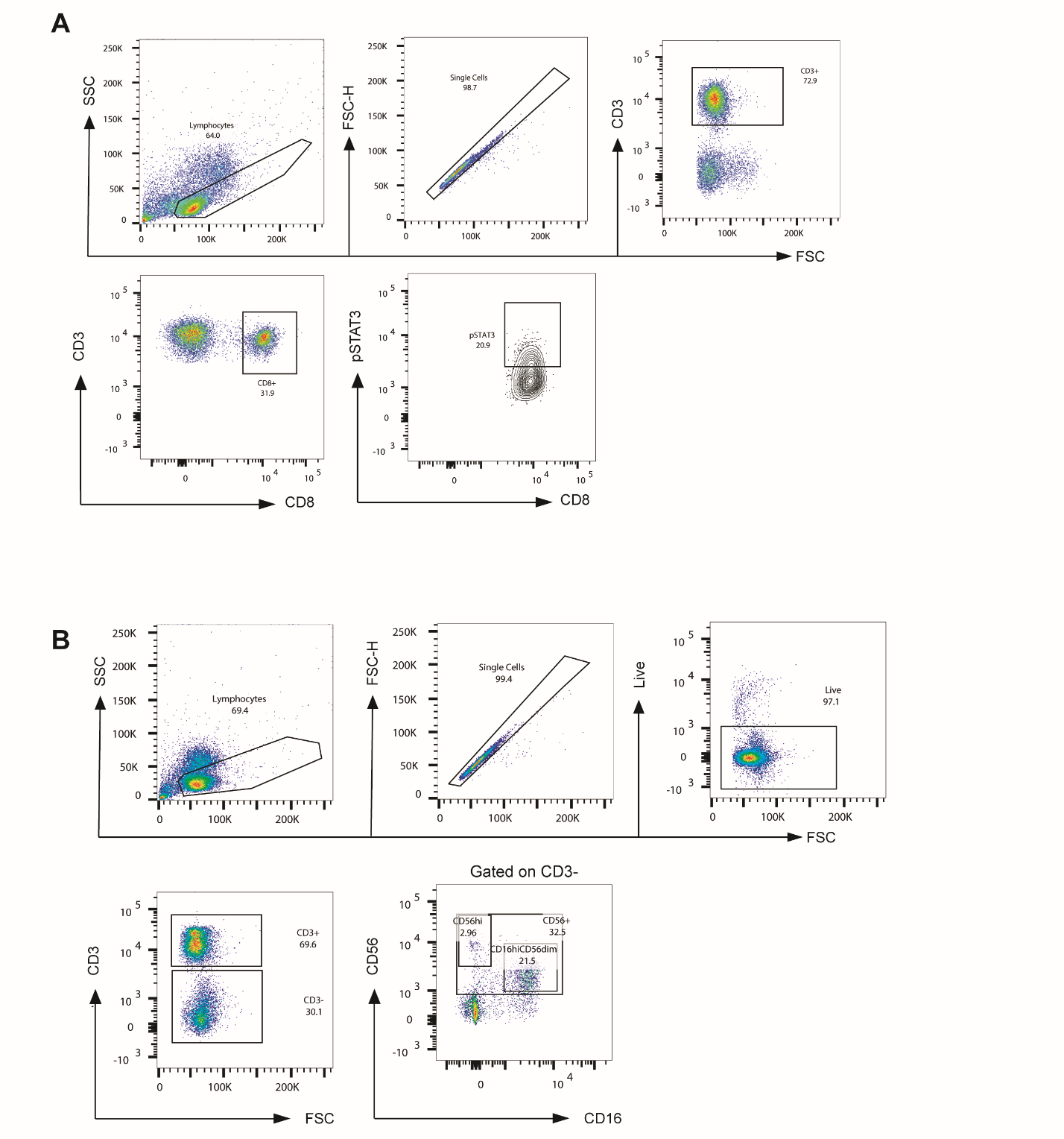


**Fig. S10**: **T and NK cell flow cytometry gating strategies.** Gates are shown for (**A**) pSTAT3 on CD8^+^ T cells and (**B**) CD56^+^ NK cells.

| **ID** | **Race, BMI (kg/m^2^)** | **PMH** | **Disease course** | **Symptoms** | **Troponin T (ng/mL)** | **BNP (pg/mL)** | **ECG** | **Echo** | **MRI** | **Time from symptom onset to stress test and Holter monitor** |
| --- | --- | --- | --- | --- | --- | --- | --- | --- | --- | --- |
| **P1** | White, 22.9 | None | **Acute** | Chest pain, fever, headache, nausea, congestion, fatigue | 0.82 | 275 | ST elevation in inferolateral leads | NL | Dx: Myopericarditis  RVEF 47%  RVEDVI 123 mL/m^2^  LVEF 57%  LVEDVI 111 mL/m^2^  Edema +  LGE +  Pericardial involvement + | NA |
|  |  |  |  |  |  |  |  |  |  |  |
|  |  |  |  |  |  |  |  |  |  |  |
|  |  |  | **Recovered** | x1 exertional syncope episode, ED visit | < 0.01 | < 50 | Slow sinus arrhythmia, ST elevation | NL | RVEF 51%  RVEDVI 115 mL/m^2^  LVEF 56%  LVEDVI 117 mL/m^2^  Edema –  T1 952 msec (1.5T)  ECV 25%  LGE +  Pericardial involvement – | Stress test:  26 wks- NL,  53 wks- NL  Holter monitor:  4 wks- NL,  13 wks- NL,  26 wks- NL |
|  |  |  |  |  |  |  |  |  |  |  |
|  |  |  |  |  |  |  |  |  |  |  |
| **P2** | White,  25.0 | None | **Acute** | Chest pain, fever, headache, myalgia | 24.50 | 429 | ST elevation, diffuse | Borderline LVEF: 50-55% | Dx: Sub-acute Myopericarditis  RVEF 53%  RVEDVI 88 mL/m^2^  LVEF 60%  LVEDVI 95 mL/m^2^  Edema –  T1 1234 msec (3T)  LGE –  Pericardial involvement + | NA |
|  |  |  |  |  |  |  |  |  |  |  |
|  |  |  |  |  |  |  |  |  |  |  |
|  |  |  | **Recovered** | Intermittent dull chest pain | < 0.01 | 20 | Nonspecific T wave abnormality | NL | RVEF 49%  RVEDVI 89 mL/m^2^  LVEF 56%  LVEDVI 92 mL/m^2^  Edema –  T1 981 msec (1.5T)  ECV 25%  LGE +  Pericardial involvement + | Stress test:  36 wks- NL  Holter monitor:  12 wks- NL,  35 wks- NL |
|  |  |  |  |  |  |  |  |  |  |  |
|  |  |  |  |  |  |  |  |  |  |  |
| **P3** | Black, 31.1 | Obesity, seasonal allergies | **Acute** | Chest pain, SOB, diaphoresis | 0.27 | < 50 | ST elevation, left axis deviation | NL | Dx: Acute myopericarditis  RVEF 58%  RVEDVI 80 mL/m^2^  LVEF 65%  LVEDVI 72 mL/m^2^  Edema +  LGE +  Pericardial involvement + | NA |
|  |  |  |  |  |  |  |  |  |  |  |
|  |  |  |  |  |  |  |  |  |  |  |
|  |  |  | **Recovered** | Asymptomatic | < 0.01 | < 50 | NL | NL | RVEF 48%  RVEDVI 92 mL/m^2^  LVEF 59%  LVEDVI 87 mL/m^2^  Edema –  T1 984 msec (1.5T)  ECV 24%  LGE +  Pericardial involvement + | Stress test:  17 wks- NL  Holter monitor:  12 wks- NL |
|  |  |  |  |  |  |  |  |  |  |  |
|  |  |  |  |  |  |  |  |  |  |  |
| **P4** | White, 27.4 | Asthma, eczema, seasonal allergies | **Acute** | Chest pain | 0.71 | 214 | ST elevation, diffuse | NL | Dx: Negative  RVEF 55%  RVEDVI 99 mL/m^2^  LVEF 58%  LVEDVI 110 mL/m^2^  Edema –  LGE –  Pericardia involvement – | NA |
|  |  |  |  |  |  |  |  |  |  |  |
|  |  |  |  |  |  |  |  |  |  |  |
|  |  |  | **Recovered** | Asymptomatic | < 0.01 | NA | NL | NL | RVEF 54%  RVEDVI 102 mL/m^2^  LVEF 57%  LVEDVI 98 mL/m^2^  Edema –  T1 984 msec (1.5T)  LGE –  Pericardial involvement – | NA |
|  |  |  |  |  |  |  |  |  |  |  |
|  |  |  |  |  |  |  |  |  |  |  |
| **P5** | Hispanic, 19.8 | None | **Acute** | Chest pain, muscle aches, sore throat, dry cough, fever, palpitations, belching, emesis | 3.33 | 978 | ST elevation | NL | Dx: Sub-acute Myopericarditis  RVEF 50%  RVEDVI 112 mL/m^2^  LVEF 58%  LVEDVI 105 mL/m^2^  Edema –  T1 >1300 msec (3T)  ECV 34%  LGE +  Pericardial involvement + | NA |
|  |  |  |  |  |  |  |  |  |  |  |
|  |  |  |  |  |  |  |  |  |  |  |
|  |  |  | **Recovered** | Asymptomatic | < 0.01 | < 50 | NL | NL | RVEF 50%  RVEDVI 206 mL/m^2^  LVEF 57%  LVEDVI 98 mL/m^2^  Edema –  T1 936 msec (1.5T)  ECV 24%  LGE +  Pericardial involvement – | Stress test:  23 wks- NL  Holter monitor:  9 wks- NL |
|  |  |  |  |  |  |  |  |  |  |  |
|  |  |  |  |  |  |  |  |  |  |  |
| **P6** | White, 38.9 | Obesity | **Acute** | Chest pain, fever, chills, fatigue, diaphoresis, emesis | 2.25 | 1,310 | Nonspecific ST wave changes, left axis deviation | Mild hypokinesis | Dx: Sub-acute myopericarditis  RVEF 59%  RVEDVI 82 mL/m^2^  LVEF 61%  LVEDVI 77 mL/m^2^  Edema –  LGE +  Pericardial involvement + | NA |
|  |  |  |  |  |  |  |  |  |  |  |
|  |  |  |  |  |  |  |  |  |  |  |
|  |  |  | **Recovered** | Asymptomatic | < 0.01 | NA | NL | NL | RVEF 52%  RVEDVI 87 mL/m^2^  LVEF 58%  LVEDVI 86 mL/m^2^  Edema –  T1 1040 msec (1.5T)  ECV 25%  LGE +  Pericardial involvement + | Stress test:  19 wks- NL  Holter monitor:  2 wks- ABN with occasional polymorphic PVCs, 8 wks- ABN with occasional PVCs,  18 wks- NL with rare PVCs |
|  |  |  |  |  |  |  |  |  |  |  |
|  |  |  |  |  |  |  |  |  |  |  |
| **P7** | White, 23.1 | Migraine without aura | **Acute** | Chest pain, nausea, malaise, fever, chills, palpitation, SOB, pre- syncope | 0.47 | 115.9 | NL | NL | Dx: Sub-acute myocarditis  RVEF 51%  RVEDVI 56 mL/m^2^  LVEF 52%  LVEDVI 79 mL/m^2^  Edema –  T1 1273 msec (3T)  ECV 40%  LGE +  Pericardial involvement + | NA |
|  |  |  |  |  |  |  |  |  |  |  |
|  |  |  |  |  |  |  |  |  |  |  |
|  |  |  | **Recovered** | SOB on excretion | < 0.01 | NA | NL | NL | RVEF 50%  RVEDVI 73 mL/m^2^  LVEF 50%  LVEDVI 83 mL/m^2^  Edema –  T1 1095 msec (1.5T)  ECV 34%  LGE +  Pericardial involvement + | Stress test:  21 wks- NL,  28 wks- NL,  53 wks- NL  Holter monitor:  8 wks- ABN with very rare PACs, PVCs,  53 wks- NL |
|  |  |  |  |  |  |  |  |  |  |  |
|  |  |  |  |  |  |  |  |  |  |  |
| **P8** | White, 31.9 | Obesity, Benign joint hypermobility syndrome | **Acute** | Chest pain, SOB, fever | 0.40 | 584 | Nonspecific ST segment and T wave abnormality | Borderline low LVEF ~50-55% | Dx: Pericarditis  RVEF 55%  RVEDVI 101 mL/m^2^  LVEF 59%  LVEDVI 96 mL/m^2^  Edema –  T1 1014 msec (1.5T)  ECV 24%  LGE –  Pericardial involvement + | NA |
|  |  |  |  |  |  |  |  |  |  |  |
|  |  |  |  |  |  |  |  |  |  |  |
|  |  |  | **Recovered** | x1 chest pain, ED visit | < 0.01 | NA | NL | Improved LVEF 56% | NA | Stress test:  18 wks- NL  Holter monitor:  15 wks- NL |
|  |  |  |  |  |  |  |  |  |  |  |
|  |  |  |  |  |  |  |  |  |  |  |
| **P9** | Hispanic, 29.2 | None | **Acute** | Chest pain | 2.23 | 497 | ST elevation, short PR interval | Small effusion (<1cm) | Dx: Sub-acute myopericarditis  RVEF 44%  RVEDVI 81 mL/m^2^  LVEF 53%  LVEDVI 66 mL/m^2^  Edema +  LGE +  Pericardial involvement + | NA |
|  |  |  |  |  |  |  |  |  |  |  |
|  |  |  |  |  |  |  |  |  |  |  |
|  |  |  | **Recovered** | Asymptomatic | < 0.01 | < 50 | NL | Mild MR | RVEF 46%  RVEDVI 80 mL/m^2^  LVEF 55%  LVEDVI 64 mL/m^2^  Edema –  LGE +  Pericardial involvement – | Stress test:  4 wks- NL  Holter monitor: NA |
|  |  |  |  |  |  |  |  |  |  |  |
|  |  |  |  |  |  |  |  |  |  |  |
| **P10** | White, 24.2 | Anxiety | **Acute** | Chest pain, SOB | 0.43 | 169 | ST elevation, diffuse | NL | NA | NA |
|  |  |  |  |  |  |  |  |  |  |  |
|  |  |  |  |  |  |  |  |  |  |  |
|  |  |  | **Recovered** | Asymptomatic | < 0.01 | 59 | NL | NL | Dx: NL  RVEF 53%  RVEDVI 105 mL/m^2^  LVEF 57%  LVEDVI 103 mL/m^2^  Edema –  T1 989 msec (1.5T)  ECV 25%  LGE –  Pericardial involvement – | NA |
|  |  |  |  |  |  |  |  |  |  |  |
|  |  |  |  |  |  |  |  |  |  |  |
| **P11** | White, 21.1 | Crohn’s disease | **Acute** | Chest pain, SOB | 0.10 | 93 | Sinus bradycardia | Mildly reduced LVEF ~48-54%  Mild TR | Dx: Sub-acute myopericarditis  RVEF 38%  RVEDVI 116 mL/m^2^  LVEF 45%  LVEDVI 103 mL/m^2^  Edema –  T1 1272 msec  ECV 27%  LGE +  Pericardial involvement + | NA |
|  |  |  |  |  |  |  |  |  |  |  |
|  |  |  |  |  |  |  |  |  |  |  |
|  |  |  | **Recovered** | Asymptomatic | < 0.01 | NA | NL | NL | RVEF 43%  RVEDVI 107 mL/m^2^  LVEF 48%  LVEDVI 93 mL/m^2^  Edema –  T1 999 msec  ECV 25%  LGE –  Pericardial involvement – | Stress test:  29 wks- NL  Holter monitor:  28 wks- NL |
|  |  |  |  |  |  |  |  |  |  |  |
|  |  |  |  |  |  |  |  |  |  |  |
| **P12** | White, 28.1 | None | **Acute** | Chest pain, SOB | 0.01 | NA | Sinus rhythm with sinus arrhythmia, ST elevation | NL | NA | NA |
|  |  |  | **Recovered** | Abdominal pain | NA | NA | NL | NA | NA | NA |
| **P13** | White, 25.9 | ADHD, hydrocele | **Acute** | Chest pain, fever, cough, malaise, diaphoresis, sneezing, neck pain | 95 | 1,269 | Atrial premature complex, diffuse T wave changes | Mild septal hypokinesis, Mild MR | Dx: Myopericarditis  LVEF 63%  Edema –  LGE +  Pericardial involvement + | NA |
|  |  |  | **Recovered** | Asymptomatic | < 6 | NA | NL | Mild MR and AR | NA | Stress test: NA  Holter monitor:  1 wk- ABN with occasional PACs. |
| **P14** | White, 18.2 | None | **Acute** | Chest pain | < 0.02 (Troponin I) | NA | Borderline short PR interval, RSR prime in V1-V2 with ST elevation | NL | Dx: Pericarditis  RVEF 61%  LVEF 67%  Edema –  ECV +  LGE –  Pericardial involvement + | NA |
|  |  |  | **Recovered** | Fatigue | < 0.02 (Troponin I) | NA | NA | NA | NA | Stress test:  2 wks- ABN ECG changes in inferior leads, early and late impairment of heart rate recovery,  8 wks- ABN evidence of ischemia  Holter monitor: NA |
| **P15** | Black, 22.5 | None | **Acute** | Chest pain | 1.64 | 58 | Wolff-Parkinson-White pattern | Mild TR | Dx: Sub-acute myopericarditis  RVEF 54%  RVEDVI 105 mL/m^2^  LVEF 59%  LVEDVI 88 mL/m^2^  Edema –  T1 1244 msec (3T)  ECV 27%  LGE +  Pericardial involvement + | NA |
|  |  |  | **Recovered** | Asymptomatic | < 0.01 | NA | NL | Mild TR and MR | RVEF 58%  RVEDVI 99 mL/m^2^  LVEF 56%  LVEDVI 94 mL/m^2^  Edema –  T1 1002 msec (1.5T)  ECV 24%  LGE +  Pericardial involvement + | Stress test:  25 wks- NL  Holter monitor:  1 wk- ABN with ventricular pre-excitation throughout |
| **P16** | Hispanic,  19.4 | None | **Acute** | Chest pain | 2.28 | 524 | ST elevation | NL | Dx: Sub-acute myopericarditis  RVEF 51%  RVEDVI 79 mL/m^2^  LVEF 51%  LVEDVI 66 mL/m^2^  Edema –  T1 1059 msec (1.5T)  ECV 26%  LGE +  Pericardial involvement + | NA |
|  |  |  | **Recovered** | Asymptomatic | < 0.01 | 94 | NL | NL | RVEF 46%  RVEDVI 86 mL/m^2^  LVEF 49%  LVEDVI 78 mL/m^2^  Edema –  T1 1000 msec (1.5T)  ECV 28%  LGE +  Pericardial involvement – | Stress test:  29 wks- NL  Holter monitor:  9 wks- NL |
| **P17** | Hispanic,  23.1 | None | **Acute** | Chest pain, palpitation | 0.32 | 487 | ST elevation | Mild global decreased LVEF 45-50% | Dx: Myocarditis  RVEF 50%  RVEDVI 84 mL/m^2^  LVEF 49%  LVEDVI 40 mL/m^2^  Edema +  LGE +  Pericardial involvement – | NA |
|  |  |  | **Recovered** | Asymptomatic | NA | NA | NA | NA | RVEF 50%  RVEDVI 86 mL/m^2^  LVEF 53%  LVEDVI 82 mL/m^2^  Edema –  LGE +  Pericardial involvement – | Stress test: NA  Holter monitor:  13 wks- NL |
| **P18** | White, 21.3 | Asthma, seasonal allergies, migraines | **Acute** | Chest pain, fever, headache, lower back pain, malaise | 2.32 | 1,344 | Borderline ST elevation | NL | Dx: Acute myocarditis  RVEF 44%  RVEDVI 96 mL/m^2^  LVEF 44%  LVEDVI 104 mL/m^2^  Edema +  LGE +  Pericardial involvement + | NA |
|  |  |  |  |  |  |  |  |  |  |  |
|  |  |  |  |  |  |  |  |  |  |  |
|  |  |  | **Recovered** | Asymptomatic | < 0.01 | < 50 | NL | NL | RVEF 44%  RVEDVI 94 mL/m^2^  LVEF 48%  LVEDVI 90 mL/m^2^  Edema –  T1 1033 msec (1.5T)  ECV 33%  LGE +  Pericardial involvement + | Stress test:  18 wks- NL  Holter monitor:  16 wks- NL |
|  |  |  |  |  |  |  |  |  |  |  |
|  |  |  |  |  |  |  |  |  |  |  |
|  |  |  |  |  |  |  |  |  |  |  |
| **P19** | Eastern European/ Asian, 25.8 | ADHD | **Acute** | Chest pain | 60.1 (Troponin I) | 39.7 (BNP) | ST elevation | Diffuse LV dysfunction LVEF 32% | Dx: Myocarditis  RVEF 48%  RVEDVI 89 mL/m^2^  LVEF 50%  LVEDVI 100 mL/m^2^  Edema –  LGE +  Pericardial involvement + | NA |
|  |  |  | **Recovered** | Asymptomatic | NA | NA | NL | NA | RVEF 51%  RVEDVI 88 mL/m^2^  LVEF 57%  LVEDVI 73 mL/m^2^  Edema –  LGE –  Pericardial involvement – | NA |
| **P20** | Black, 29.1 | Asthma | **Acute** | Chest pain, dyspnea on exertion, fever, vomiting, diarrhea | 61.3 (Troponin I) | 138.8 | ST elevation | Global mild-to-moderate LV systolic dysfunction; low normal RV systolic function | Dx: Myocarditis  RVEF 39%  RVEDVI 119 mL/m^2^  LVEF 44%  LVEDVI 101 mL/m^2^  Edema –  LGE +  Pericardial involvement – | NA |
|  |  |  | **Recovered** | x2 chest pain, ED visit | < 0.01 | NA | Slight T-wave inversion | NL | RVEF 51%  RVEDVI 88 mL/m^2^  LVEF 57%  LVEDVI 73 mL/m^2^  Edema –  LGE –  Pericardial involvement – | NA |
| **P21** | White, 22.8 | Anxiety, depression | **Acute** | Chest pain, SOB, body aches, general weakness, productive cough, nasal congestion | < 0.01 | NA | NL | NL | Dx: Myocarditis  RVEF 59%  RVEDVI 88 mL/m^2^  LVEF 58%  LVEDVI 86 mL/m^2^  Edema –  LGE +  Pericardial involvement – | NA |
|  |  |  | **Recovered** | Asymptomatic | NA | NA | NA | NA | RVEF 61%  RVEDVI 82 mL/m^2^  LVEF 60%  LVEDVI 83 mL/m^2^  Edema –  LGE +  Pericardial involvement – | Stress test:  14 wks- NL  Holter monitor:  14 wks- NL |
| **P22** | Black, 32.9 | Obesity, autism, ADHD | **Acute** | Chest pain, cough, throat pain, decreased appetite, fatigue, headache | 1.42 | 1,483 | Sinus rhythm with complete AV block, escape rate 48 bpm with wide complex QRS | NL | Dx: Myocarditis  RVEF 54%  RVEDVI 68 mL/m^2^  LVEF 53 %  LVEDVI 73 mL/m^2^  Edema –  LGE +  Pericardial involvement – | NA |
|  |  |  | **Recovered** | Asymptomatic | < 0.01 | < 50 | NL | NL | RVEF 43%  RVEDVI 74 mL/m^2^  LVEF 47%  LVEDVI 73 mL/m^2^  Edema –  T1 1017 msec (1.5T)  LGE +  Pericardial involvement + | Stress test:  30 wks- NL  Holter monitor:  2 wks- NL,  32 wks- ABN with occasional PVCs |
| **P23** | White,  22.6 | Asthma, allergic rhinitis, anxiety, speech delay | **Acute** | Fever, chills, headache, emesis, diarrhea | < 0.01 | 4,687 | ST elevation | NL | NA | NA |
|  |  |  | **Recovered** | Asymptomatic | < 0.01 | 53 | NL | NL | NA | Stress test:  9 wks- NL  Holter monitor: NA |

**Table S1. Clinical characteristics of the myopericarditis patient cohort during acute illness and follow-up/recovery.** BMI-body mass index; PMH-past medical history; BNP-B-type natriuretic peptide; ECG-electrocardiogram; Echo-echocardiogram; MRI-magnetic resonance imaging; SOB-shortness of breath; ED-emergency department; Dx-diagnosis; RVEF-right ventricular ejection fraction; RVEDVI-right ventricular end diastolic volume index; LVEF-left ventricular ejection fraction; LVEDVI-left ventricular end diastolic volume index; LGE-late gadolinium enhancement; ECV-extracellular volume; mL/m^2^-milliliter per square meter; msec-millisecond; LV-left ventricle, RV-right ventricle; AV-atrioventricular; MR-mitral regurgitation; TR-tricuspid regurgitation; AR-aortic regurgitation; bpm-beats per minute; PVC-premature ventricular contraction; PAC-premature atrial complex; wks-weeks; wk-week; NL-normal; NA-not applicable; ABN-abnormal.

| **ID** | **Sex** | **Vaccine type (dose)** | **Acute**  **Clinical Labs** | **SARS-CoV-2 Abs** | **Neutralization** | **REAP aAbs** | **Cytokines/**  **proteomics** | **scRNA-seq/**  **repertoire** | **Flow cytometry/ ELISA**  **(IL-15/**  **sCD163)** |
| --- | --- | --- | --- | --- | --- | --- | --- | --- | --- |
| **Myopericarditis Patients**  **Age 13-21 (16.9 +/- 2.2)** | | | | | | | | | |
| **P1** | M | Pfizer (2) | 3 days | 3 days | 3 days | 3 days | 3 days | 4 days | 4 days |
| **P2** | M | Pfizer (2) | 3 days | 4 days | 4 days | 4 days | 4 days | 5 days | 5 days |
| **P3** | M | Pfizer (2) | 3 days | 3 days | 3 days | 3 days | 3 days | 5 days | 5 days |
| **P4** | M | Pfizer (2) | 3 days | 3 days | 3 days | 3 days | 3 days | 4 days | 4 days |
| **P5** | M | Pfizer (2) | 10 days | 11 days | 11 days | 11 days | 11 days |  |  |
| **P6** | M | Pfizer (2) | 3 days | 3 days | 3 days | 3 days | 3 days |  |  |
| **P7** | F | Pfizer (2) | 3 days | 3 days | 3 days | 3 days | 3 days |  |  |
| **P8** | M | Pfizer (2) | 2 days | 3 days | 3 days | 3 days | 3 days |  |  |
| **P9** | M | Pfizer (2) | 2 days | 4 days | 4 days | 4 days | 4 days |  |  |
| **P10** | M | Pfizer (2) | 2 days |  |  |  |  |  |  |
| **P11** | M | Pfizer (2) | 3 days |  | 4 days |  |  |  |  |
| **P12** | M | Moderna (2) | 5 days |  |  |  |  |  |  |
| **P13** | M | Pfizer (3) | 3 days |  |  |  |  |  |  |
| **P14** | M | Moderna (2) | 1 day |  |  |  |  |  |  |
| **P15** | M | Pfizer (2) | 4 days |  |  |  |  |  |  |
| **P16** | M | Pfizer (2) | 4 days |  |  |  |  |  |  |
| **P17** | F | Pfizer (2) | 3 days |  |  |  |  |  |  |
| **P18** | M | Moderna (1) | 21 days |  |  |  |  |  |  |
| **P19^** | M | Moderna (3) | 2 days |  |  |  |  |  |  |
| **P20** | M | Pfizer (2) | 30 days |  |  |  |  |  |  |
| **P21^** | F | Pfizer (3) | 26 days |  |  |  |  |  |  |
| **P22** | M | Pfizer (2) | 31 days |  |  |  |  |  |  |
| **P23** | M | Pfizer (1) | 28 days |  |  |  |  |  |  |
| **Early-Young Vaccinated (three doses) Healthy Donors**  **Age 20-21 (20.3 +/- 0.5)** | | | | | | | | | |
| **E-YVC1** | M | Pfizer |  |  |  |  |  | 3 days | 3 days |
| **E-YVC2** | M | Moderna |  |  |  |  |  | 5 days | 5 days |
| **E-YVC3** | M | Pfizer |  |  |  |  |  | 5 days | 5 days |
| **E-YVC4** | M | Pfizer |  |  |  |  |  | 2 days | 2 days |
| **Unvaccinated Pediatric Healthy Donors**  **Age 6-14 (9.8 +/- 3.3)** | | | | | | | | | |
| **HD1** | M | NA |  |  |  |  |  | x | x |
| **HD2** | M | NA |  |  |  |  |  | x | x |
| **HD3** | M | NA |  |  |  |  |  | x | x |
| **HD4** | M | NA |  |  |  |  |  | x | x |
| **Vaccinated (two doses) Healthy Donors**  **Age 26-67 (40 +/- 11.4); YVC Age 26-29 (28 +/- 1.2); All 7 days after vaccination** | | | | | | | | | |
| **VC1** | F | Pfizer |  | x | x |  |  |  |  |
| **VC2** | M | Pfizer |  | x | x |  |  |  |  |
| **VC3** | F | Pfizer |  | x | x |  |  |  |  |
| **VC4** | F | Pfizer |  | x | x |  |  |  |  |
| **VC5** | F | Pfizer |  | x | x |  |  |  |  |
| **VC6** | F | Pfizer |  | x | x |  |  |  |  |
| **VC7** | F | Moderna |  | x | x |  |  |  |  |
| **VC8** | M | Moderna |  | x | x |  |  |  |  |
| **VC9** | F | Moderna |  | x | x |  |  |  |  |
| **VC10** | F | Moderna |  | x | x |  |  |  |  |
| **VC11** | F | Moderna |  | x | x |  |  |  |  |
| **VC12** | F | Moderna |  | x | x |  |  |  |  |
| **VC13** | M | Moderna |  | x | x |  |  |  |  |
| **VC14** | F | Moderna |  | x | x |  |  |  |  |
| **VC15** | F | Moderna |  | x | x |  |  |  |  |
| **VC16** | F | Moderna |  | x | x |  |  |  |  |
| **YVC1** | F | Moderna |  | x | x | x | x |  |  |
| **YVC2** | M | Moderna |  | x | x | x | x |  |  |
| **YVC3** | F | Moderna |  | x | x | x | x |  |  |
| **YVC4** | M | Moderna |  | x | x | x | x |  |  |
| **YVC5** | F | Pfizer |  | x | x | x | x |  |  |
| **YVC6** | M | Pfizer |  | x | x | x | x |  |  |

**Table S2. Patients and control samples used for different assays.** Sex, vaccine type and dose, as well as days after last vaccination dose at which blood samples were collected for each assay. ^ = SARS-CoV-2 PCR + patients (incidental at admission).

**Table S3. CITE-seq panel.** List of the 189 surface protein markers, corresponding genes, and associated sequences. Separate Excel spreadsheet.
